# Clusters of Glycemic Response to Oral Glucose Tolerance Tests Explain Multivariate Metabolic and Anthropometric Outcomes of Bariatric Surgery in Obese Patients

**DOI:** 10.3390/jcm8081091

**Published:** 2019-07-24

**Authors:** Lukasz Szczerbinski, Mark A. Taylor, Anna Citko, Maria Gorska, Steen Larsen, Hady Razak Hady, Adam Kretowski

**Affiliations:** 1 Department of Endocrinology, Diabetology and Internal Medicine; Medical University of Bialystok, Sklodowskiej-Curie 24A, 15-276 Bialystok, Poland; 2 School of Medicine, University of California at San Francisco, 505 Parnassus Ave., San Francisco, CA 94143, USA; 3 Clinical Research Centre; Medical University of Bialystok, Sklodowskiej-Curie 24A, 15-276 Bialystok, Poland; 4 Department of Biomedical Sciences; University of Copenhagen, Blegdamsvej 3, 2200 Copenhagen N, Denmark; 5 1st Clinical Department of General and Endocrine Surgery; Medical University of Bialystok, Sklodowskiej-Curie 24A, 15-276 Bialystok, Poland

**Keywords:** bariatric surgery, diabetes, glucose homeostasis, latent trajectory

## Abstract

Glycemic responses to bariatric surgery are highly heterogeneous among patients and defining response types remains challenging. Recently developed data-driven clustering methods have uncovered subtle pathophysiologically informative patterns among patients without diabetes. This study aimed to explain responses among patients with and without diabetes to bariatric surgery with clusters of glucose concentration during oral glucose tolerance tests (OGTTs). We assessed 30 parameters at baseline and at four subsequent follow-up visits over one year on 154 participants in the Bialystok Bariatric Surgery Study. We applied latent trajectory classification to OGTTs and multinomial regression and generalized linear mixed models to explain differential responses among clusters. OGTT trajectories created four clusters representing increasing dysglycemias that were discordant from standard diabetes diagnosis criteria. The baseline OGTT cluster increased the predictive power of regression models by over 31% and aided in correctly predicting more than 83% of diabetes remissions. Principal component analysis showed that the glucose homeostasis response primarily occurred as improved insulin sensitivity concomitant with improved the OGTT cluster. In sum, OGTT clustering explained multiple, correlated responses to metabolic surgery. The OGTT is an intuitive and easy-to-implement index of improvement that stratifies patients into response types, a vital first step in personalizing diabetic care in obese subjects.

## 1. Introduction

Bariatric surgery is the single most effective treatment for type 2 diabetes (T2D), with a complete two year remission in over 78% of patients and marked improvement in over 86% of patients, as well as improved prediabetic status [1]. However, there remains unexplained heterogeneity in both the magnitude and durability of this intervention’s beneficial effects [2]. In particular, approximately 28% of patients with T2D do not remit two years post-surgery, and 16% are already non-obese (BMI < 30 kg/m^2^) and do not qualify for standard bariatric surgical therapy [3,4]. As obesity incidence accelerates in both the developed and developing world [5], and comorbid dysglycemias concomitantly rise, there is an increasing need for clinically tractable methods to understand and stratify response groups. Existing methods to define dysglycemic groups are largely phenomenological (i.e., not motivated by causal factors but rather by correlates) and rely upon optimizing the statistical machinery of the clustering algorithm rather than choosing variables that make pathophysiological sense [6]. Promisingly, a new class of deep analytic methods, latent trajectory classification models, have recently been implemented to detect subtle differences among patient groups that reflect metabolic and physiological mechanisms [7]. Here, we apply these methods to a group of patients both with and without diabetes in order to gain insight into differences in metabolic and anthropometric responses to bariatric surgery based upon these latent clusters. To our knowledge, this is the first time that these methods have been used for a mixed cohort of patients with and without diabetes undergoing the same bariatric surgical treatment.

The mechanistic basis of bariatric surgery’s ameliorative effect on T2D is complex and is known to be at least partially independent from its effect on weight loss [8,9,10]. Indeed, just one week post-surgery before any significant weight loss, dysglycemia features largely remit with insulin sensitivity improved, insulin output dramatically decreased, and beta-cell glucose sensitivity increased [9,11]. The mechanism of this rapid response may rest upon improved liver and pancreatic function [12], and a number of proxy measurements of their function have been identified such as alanine transaminase (ALT) concentration and HOMA-β, respectively. However, a constellation of correlated secondary effects also occurs following bariatric surgery, such as improved appetite control, decreased bile acid concentration, altered intestinal microbiota, and changes in gut hormones [13,14,15]. The direction and magnitude of causality among these correlated factors remain unclear [8,14]. The benefit of bariatric surgery is integrated across them so that it is desirable to develop methods that gauge this multivariate response. Here, we implement a principal component approach to simultaneously measure responses integrated over anthropometric, general metabolic, and glucose homeostasis parameters.

To predict diabetes responses to bariatric surgery, a number of formal predictive models have been developed. However, these models often rely upon non-mechanistic, tautological predictors, such as the time since diabetes diagnosis and medication number. Although they predict remission with high sensitivity and specificity, they are rarely used in the clinic and their mechanistic insight into diabetes remission is limited [16,17,18,19,20,21,22,23]. The lack of a mechanism in these diabetes models is likely due to the etiological complexity of diabetes—some types are predominantly associated with insulin resistance and obesity and others with diminished beta-cell function [24]. Differentiating among these subtypes is prohibitively time-consuming and expensive, requiring that multiple different tests be performed [25]. Oral glucose tolerance tests, on the other hand, represent a single, well-known approach that has been found to characterize many facets of type 2 diabetes etiology [26]. Glucose or insulin concentration curves from oral glucose tolerance tests (OGTTs) have already been successfully used to differentiate among subtypes and to predict diabetic risks [7,26,27,28]. However, these curves have never been used to explain or predict glycemic or multivariate responses to bariatric surgery.

Characterizing data taken from multi-point OGTTs is a complex problem. For example, these datasets could be modeled as linear, quadratic, or even higher order curves. From these models, which terms should be considered informative (i.e., intercepts, slopes, maxima, or minima)? The answers to these questions may not be straightforward, instead often relying upon complex and occasionally arbitrary model selection procedures [29]. However, latent class trajectory models are an attractive alternative method to accomplish the goal of analyzing OGTT curves. Latent class trajectory algorithms can uncover subtle patterns in longitudinal data that may not be obvious to observers who simply plot the data to observe patterns or even those who employ traditional discriminant analysis [30]. Latent class trajectories have already been shown to discriminate among pathophysiologically insightful groups of patients with type 2 diabetes based upon OGTT data [7], and here we apply these methods to bariatric surgical outcomes.

There were four primary aims of this study: (1) to examine how the glucose concentration curves of response to OGTT of candidates for bariatric surgery differ by modeling them as latent trajectories; (2) to determine whether these trajectories explain differences in response to bariatric surgical intervention in anthropometric and metabolic parameters; (3) to examine simultaneous responses in anthropometric and metabolic parameters by leveraging principal components; and (4) to assess the explanatory power of latent trajectory clusters in predicting diabetes improvement.

## 2. Experimental Section

### 2.1. Study Design

**Figure 1:**
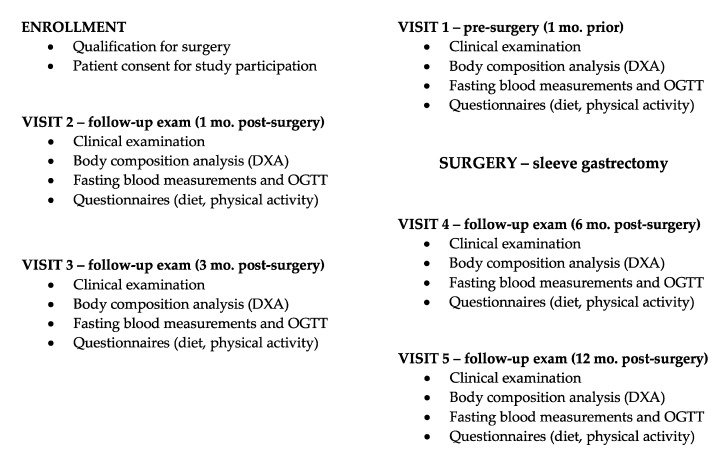
Flowchart of study design emphasizing measurements taken on each patient from enrollment to final follow-up exam 12 mo after bariatric surgery. DXA, dual energy X-ray absorptiometry; OGTT, oral glucose tolerance tests.

The Bialystok Bariatric Surgery Study (BBSS) is a prospective cohort study of patients undergoing bariatric surgery at the First Clinical Department of General and Endocrine Surgery at the Medical University of Bialystok. This is the primary receiving center for patients referred for bariatric surgery in the province of Podlaskie Voivodeship and the largest center by number of bariatric surgeries performed in northeastern Poland. This center specializes in several bariatric surgical techniques including Roux-en-Y gastric bypass, gastric banding, and sleeve gastrectomy. For this study, we selected only patients who underwent sleeve gastrectomy since it represents the vast majority (85%) of all interventions performed at the center and in order to eliminate confounding variation in surgical technique. The BBSS began in 2016 and consisted of a battery of baseline tests established one month prior to the intervention and repeated at one, three, six, and twelve month follow-up clinical visits. The study design is presented as a flowchart in Figure 1. At each visit, all subjects underwent physical examination, body composition analysis, OGTT, and blood testing, as well as completed diet and physical activity questionnaires. All subjects gave their informed consent for inclusion before participating in the study. The study was conducted in accordance with the Declaration of Helsinki, and the protocol was approved by the Ethics Committee of the Medical University of Bialystok (Project identification code: R-I-002/546/2015).

### 2.2. Study Population

The enrollment of 154 patients occurred from 2016 to 2018. In accordance with the National Institutes of Health guidelines for bariatric surgery [31], inclusion criteria consisted of a BMI ≥ 40 kg/m^2^ or a BMI ≥ 35 kg/m^2^ with comorbidities. Exclusion criteria were any prior bariatric surgery, gastrectomy, substance abuse, uncontrolled psychiatric illness, expected lack of compliance, or advanced stage cancer. Diabetes diagnoses were based on the dysglycemia diagnostic criteria of Diabetes Poland (PTD) [32] using OGTT results only at 0 and 120 min. Four patients (2.6%) presented with prediabetes, a strongly positive family history of diabetes, and were taking prophylactic antidiabetic medication, but they had never received a diagnosis of diabetes and their OGTT tests were negative for T2D. Since definitive diabetes diagnoses through history or OGTT, these patients’ diagnoses were coded as missing and not included in analyses considering diagnostic state. We defined remission of diabetes according to the American Society for Metabolic and Bariatric Surgery’s guidelines [33], which were a normal measure of glucose metabolism defined as HbA1c <6% and fasting blood glucose (FBG) <100 mg/dL in the absence of antidiabetic medication therapy.

### 2.3. Assay Protocols and Measurements

Glucose homeostasis, general metabolic (cardiovascular, lipid, and fat-tissue-related), and anthropometric parameters were measured at baseline and each follow-up exam (individual measurements listed in Table 1). The OGTT procedure was conducted in accordance with the American Diabetes Association (ADA) recommendations, with tests commencing between 7:30 and 8:00 in the morning [34]. Patients were instructed to fast for 8–10 h and avoid intensive exercise for 24h prior to the test. Patients were instructed not take any medications the day of the test and not to use bicycles or stairs in their commute to the clinic. Patients with diabetes were instructed to avoid oral antidiabetic agents for 24 h prior to the surgery. The test started with baseline blood collection (0 min), followed by oral consumption of the solution of 75 g of glucose in 300 mL of room temperature water. Next, blood collections were performed 30, 60, and 120 min after the glucose administration. We used these time measurements to construct curves that allowed the calculation of glucose area under the curve (AUC), insulin AUC, Matsuda index, mean insulin concentration, and mean glucose concentration.

Average daily energy intake (kcal/day), as well as average daily intake of proteins, carbohydrates and fat were calculated based on dietary records for the three days before each visit. Patients were instructed to maintain their average quotidian dietary habits for at least three days prior to each visit (including at least one weekend day), and compliance was monitored by analyzing their diet diaries. These data were then analyzed using Dieta 4.0 (National Food and Nutrition Institute, Warsaw, Poland), which collated and summarized nutrient type-specific (i.e., carbohydrate, protein, and fat) percent and mass intake, as well as daily energy intake. At this time, physical activity was also assessed using the Polish version of the International Physical Activity Questionnaire-Long Form (IPAQ-LF) [35]. Whole body dual energy X-ray absorptiometry (DXA) scans were performed for body composition analysis, using Lunar iDXA (GE Healthcare, Chicago, IL, USA). The total amount of lean body mass (LBM), fat mass (FM), and visceral adipose tissue (VAT) mass were measured and expressed in kilograms.

### 2.4. Statistical Analysis

Glucose response classification: We implemented a latent trajectory classification algorithm on baseline plasma glucose concentrations during the 4-point OGTT to detect glucose curve characteristics that differentiated patients into pathophysiologically relevant groups. The pathophysiology under consideration in this study was dysglycemia occurring during OGTT, so relevant groups were those that differed in characteristics of their OGTT curves. These clusters were first described by Hulman et al. in a large prospective study on the European Group for the Study of Insulin Resistance [7]. We assigned patients to the cluster for which they had the highest probability of membership, repeating this procedure in each follow-up exam for subjects with complete 4-point OGTTs. All analyzed patients had complete 4-point baseline OGTTs; 35% had complete OGTTs at every clinical encounter.

Cross sectional analyses: All summary statistics and subsequent analyses were performed in *R* [36]. Discrete parameters (i.e., smoking status, diagnosis, etc) were presented as frequencies (percentages) and continuous parameters as medians (interquartile range [IQR]). For continuous parameters, we implemented two of three possible quantile regression models to estimate median and IQR adjusted for: (1) age and sex by model 1; (2) age, sex, and smoking status by model 2; (3) age, sex, smoking status, and baseline BMI by model 3. Models 1 and 2 were used for continuous variables based upon or highly correlated to BMI (such as weight), while models 1 and 3 were used for all others. To compare whether medians among clusters differed significantly, we performed non-parametric median tests implemented from Conover, 1999 [37]. For all cross-sectional analyses, patients with missing data in the response variable were excluded, and no imputation was performed since sample sizes were too small and distributions too unstable to have high confidence in imputed values.

For each response parameter, we fit a repeated measures generalized linear mixed model under gamma distributions with both linear and quadratic terms for time with patient ID, smoking status, and sex fit as random effects. Model selection via hierarchical model selection compared linear and quadratic models to determine whether a linear or quadratic time term was more significantly informative. These tests showed that the quadratic models were significantly more informative, so were retained in downstream analyses (Table S1). To determine when during the time-series a parameter became significantly different from baseline, we fit generalized linear mixed models under gamma distributions accounting for diet (total kcal) and exercise (total METs-minutes/week) changes testing for post-hoc differences among follow-up exams, adjusting for multiple testing by Bonferroni corrections.

To calculate joint responses in the three parameter categories of glucose homeostasis, general metabolic, and anthropometric parameters (Table 1), we used a principal components analysis to decompose the scaled change from the baseline to the 12 month follow-up in each parameter category into two dimensions. We then plotted subjects’ PC scores along the first two eigenvectors within each eigenspace, resulting in 6 eigenvalues for each subject (2 for each of the 3 parameter categories). We used generalized linear mixed models fit to determine whether glucose response categories could discriminate among subjects within this 6-dimensional eigenspace.

OGTT cluster as predictor: To estimate how much variation diagnosis and OGTT clustering can explain in quantitative responses, we calculated a generalized marginal *R*^2^ for the fixed effects of mixed models incorporating age, sex, and smoking status [38]. To determine which parameters predicted diabetes remission among patients, we used nested logistic regression for increasingly parameterized models beginning with patient history parameters since they are commonly in use, adding the glucose OGTT cluster and ending with the most parsimonious model that correctly predicted all remission events. The models contained the following baseline terms: Model A with patient history variables (BMI, number of past years smoking, and years since diabetes diagnosis); Model B included patient history variables and the OGTT cluster; Model C included patient history variables, OGTT cluster, C reactive protein concertation, and HbA1c; and Model D included with patient history variables, the OGTT cluster, C reactive protein concentration, HbA1c, and the the number of medications prescribed to a patient one week prior to surgery considering only statins, metformin, gliptins, sulfonylureas, and gliflozins. From these models, we calculated the area-under-the-curve (AUC) of receiver operated characteristic (ROC) plots as metrics of their predictive performance.

## 3. Results

### 3.1. Study Cohort Characteristics

Of 154 enrolled patients, 138 were retained based upon clustering likelihood and with OGTT plasma glucose concentrations measurements at 3 or 4 follow-up visits. Baseline clinical characteristics of the study cohort consisting of 63 males and 75 females divided by OGTT cluster are described in Table 2. Across clusters, 13 (9.7%) were non-diabetic; 54 (40.3%) showed impaired fasting glucose (IFG) without impaired glucose tolerance; 26 (19.4%) showed both; 20 (14.9%) had newly diagnosed, untreated type 2 diabetes; and 21 (15.7%) had previously diagnosed diabetes and were undergoing oral drug therapy (Table S2). The median age (IQR) was 46 years (38–54 years) and median BMI at baseline was 45.1 kg/m^2^ (41.7–49.7 kg/m^2^). 59 (43%) participants reported no history of smoking; 59 (43%) previously smoked but quit before enrollment; and 20 (14%) smoked during the study period. The median baseline physical activity reported by IPAQ was 5061 (2565-11970) MET-min/week, and average daily calorie intake at baseline was 1714 kcal (1342–2227 kcal). Food diaries showed a median daily intake of 76.4 g of protein (58.2–100.0 g); 56.4 g of mixed fats (40.2–77.1 g); and 241.4 g of simple and complex carbohydrates (183.6–314.6 g).

### 3.2. Baseline Glucose Response Clusters

**Figure 2:**
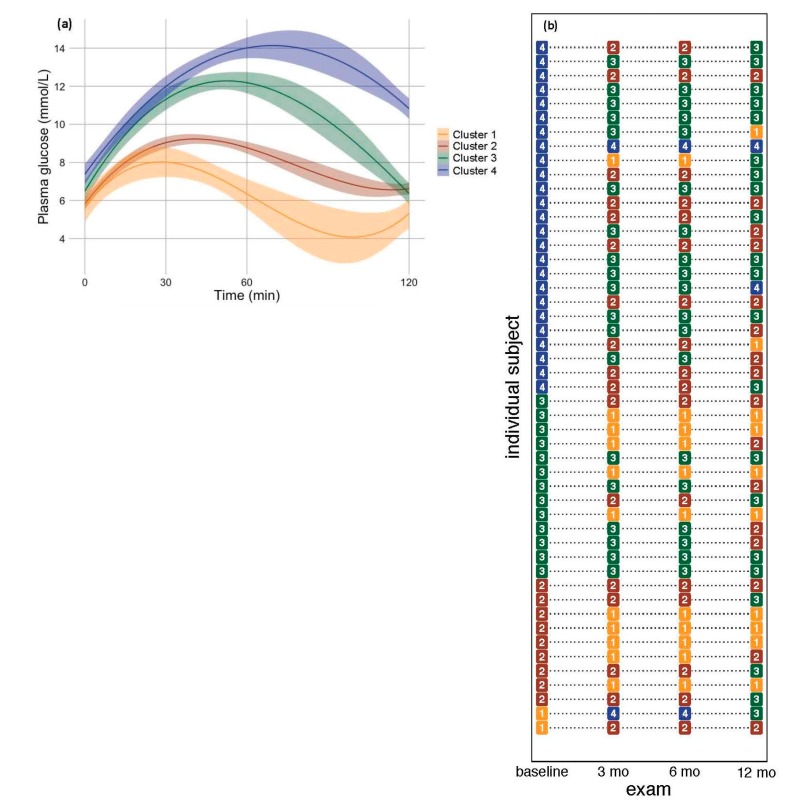
(**a**) Baseline latent OGTT glucose response trajectories fit with a third-order polynomial smoothing function. The shaded area represents 95% confidence intervals. (**b**) OGTT clusters for 48 (35.1% of total patients) with sufficient OGTT response data to classify the entire study period.

The optimal fit of the latent trajectory model to the OGTTs classified subjects into four categories which we termed Clusters 1-4 after Hulman et al. 2019 [7]. These clusters formed a discrete gradient of increasingly pathological glucose homeostasis (Figure 2a). Cluster 1 represented the most efficient glucose clearing from the blood, peaking at 30 min post-bolus and returning to initial concentration by 120 min. Cluster 1 was the smallest cross-section (*n* = 6) of the study cohort, likely due to the glycemic dysregulation inherent in a cohort selected for bariatric surgery (Table S2). Cluster 2 represented patients (*n* = 37) whose glucose load also peaked at 30 min but which returned to slightly higher values than the initial values. Clusters 3 and 4 represented severe glucose homeostasis dysregulation. Cluster 3 (*n* = 31) represented high glucose load spikes at 30 and 60 min intervals but which returned close to the initial values, and Cluster 4 (*n* = 64) represented even higher glucose spikes that did not resolve within the OGTT’s 120 min study window.

These clusters were distinct from a prediabetes or diabetes diagnosis (Table S2). For example, Clusters 3 and 4 occurred in patients with diabetes, and did not correlate to pharmacological treatment since they also occurred in newly diagnosed, untreated patients. Furthermore, all four clusters occurred in those with impaired fasting glucose (IFG), and Clusters 2, 3, and 4 occurred in those with IFG and impaired glucose tolerance (IGT). Together, this result indicates that these clusters may detect latent information within glucose response patterns that are not captured by the standard criteria for dysglycemia diagnosis.

Comparing cluster-specific medians of baseline values showed that clusters significantly differentiated glucose homeostasis parameters prior to surgery but not anthropometric parameters except for lean body mass. Specifically, insulin AUC and mean concentrations during OGTT were lower in Clusters 1 and 2 compared to Clusters three and four. Summaries of glucose loads, like glucose AUC and mean concentration during OGTT, were lowest in Cluster 1 and increased to Cluster 4. HbA1c was also lowest in Cluster 1 and highest in Cluster 4, indicating that the extremely short-term fluctuations in glucose concertation upon which OGTT clusters were based could also predict longer term glucose loads integrated by HbA1c. Triglycerides also generally followed this increasing pattern with cluster number, and for lean body mass, Cluster 1 was greater than the other clusters. Model adjusted estimates are presented in supplementary Table S3, and no estimate was more than 15% different than its arithmetic counterpart.

### 3.3. OGTT Clustering in Response to Bariatric Surgery

To understand how bariatric surgery affects glucose response classification, we classified each subject’s OGTT curve individually at baseline and at three subsequent follow-up exams for 48 (35% of the total) subjects who had complete OGTT curves at each follow-up visit (Figure 2b). The small sample size for patients that were Cluster 1 at baseline (*n* = 2) makes patterns difficult to discern, but in the others, there were clear patterns of improvement by decreasing the cluster after bariatric surgery. Specifically, five (56%) of the baseline Cluster 2 subjects displayed a Cluster 1 OGTT curve at some point after surgery, and four (44%) of these were stable over the entire follow-up period. Of baseline Cluster 3 subjects, 9 (69%) showed an improved glucose response classification by 12 mo after surgery by a mixture of Cluster 1 and 2, and four of these were Cluster 1. Of the baseline Cluster 4 subjects, 24 (92%) showed improved classification, and only two remained unchanged. However, of these 24, only two returned to the “healthiest” Cluster 1 glucose response curve. Together, these results indicate that a complete resolution of glucose response measured in these OGTT curve classifications depends upon the severity of the initial dysregulation—the majority of the higher baseline clusters improved only partially to Clusters 2 or 3 at 12 mo post-surgery.

### 3.4. Individual Parameter Responses

In order to understand how and for whom bariatric surgery was beneficial, we fit mixed effects models to cohort-wide (all patients) and cross-sectional (OGTT cluster-specific) longitudinal responses in each measured parameter to bariatric surgery. For the cohort-wide sample, we first asked whether and when bariatric surgery was effective. All anthropometric measurements showed immediate and significant improvements in the first follow-up exam 1 mo after surgery (Table S4), which were sustained over the entire follow-up period. Across anthropometric variables, there was an average improvement of 26.8% from baseline to the 12 mo follow-up (the average of the model-adjusted means (final–baseline) / baseline) ranging from a 7.2% reduction in waist-to-hip ratio to a 56.0% decrease in visceral adipose tissue mass (Table S5). Glucose homeostasis parameters also improved one month following surgical intervention, but, although these decreases were sustained over the twelve month study period, they did not continue to decrease at the same rate as the anthropometric measurements. On average, glucose homeostasis parameters improved by 68.1% from the baseline, ranging from an 11.4% decrease in HbA1c to a >200% decrease in the Matsuda index. General metabolic parameters showed more complex and attenuated changes relative to the other variable categories, though most changed significantly by the first or second follow-up exams (1 and 3 mo, respectively). On average, the general metabolic parameters changed by 28.9% from baseline ranging from a 2.3% decrease in LDL cholesterol to a 53.8% decrease in C-reactive protein. Physical activity significantly increased by the second follow-up (3 mo) and by the end had increased by 52.4%.

### 3.5. One Year Temporal Response pattern to Bariatric Surgery

Model selection revealed that a quadratic relationship between time and the majority of response parameters was more significantly informative than a linear relationship (Table S1), and most curves were decelerating. Clinically, this indicates that the patients’ response to bariatric surgery plateaued, i.e., that the benefit likely did not continue to accumulate beyond the final follow-up visit. This shows that the twelve month window of assessment following surgery was sufficient to capture the bulk of patients’ responses such that it is unlikely that major responses emerged after twelve months of follow-up.

### 3.6. Explanatory Power of Glucose Response Classification

A major question of this study focused on whether OGTT clusters could be used to better explain the outcomes of bariatric surgery. Since patients often present with diagnoses, we first used diabetes diagnosis only; then, the glucose response cluster only; and, finally, both. We implemented a mixed effects model with three covariates that are commonly ascertained in clinical settings: age, sex, and smoking history. By including the OGTT cluster as a predictor in these models, we improved their explanatory power relative to models with only diagnosis by an average of: (1) 11% for anthropometric variables; (2) 25.9% for glucose homeostasis variables; and (3) 38.5% for general metabolic variables. The greatest absolute increase in explanatory power occurs among glucose homeostasis parameters in with *R*^2^ increases from 0.32 to 0.41, and, despite the smaller relative increase, together these parameters are able to explain almost half of the variation in the response of glucose homeostasis parameters to bariatric surgery (Table 3).

### 3.7. Integrated Multiple Parameter Responses: Principal Responses

Since bariatric surgery causes changes in many anthropometric and glucose homeostasis parameters, we wished to analyze their responses simultaneously. There were a number of cluster-specific responses for individual parameters (Table S6). For example, Cluster 4 exhibited a significant decrease in HbA1c, whereas the other clusters were not significantly different from the others when comparing the baseline to the final visit (Table S6). Any one metric represents only one response axis, but it is desirable to understand a patient’s movement along all health axes simultaneously and to weigh the relative improvement among different axes. To do this, we decomposed anthropometric and glucose homeostasis scaled changes into principal components (PCs). These PCs are the major axes of variation in the response data, i.e., the direction of the greatest amount of durable change integrating across anthropometric parameters or glucose homeostasis parameters. These PC’s form new glucose homeostasis or anthropometric measurements that integrate all of the individual metrics into a novel, data-driven health index.

The first PC for glucose homeostasis explained more than half (51.5%) of all of the variation in the glucose homeostasis data, largely representing responses in HOMA-IR and estimates of the Matsuda index (Figure S1). These are both measurements of insulin sensitivity. The second PC represents the most distinct response relative to the first PC and explains 26.1% of the variation in the glucose homeostasis data. The second PC largely represents responses in glycated hemoglobin (i.e., HbA1c loads most strongly onto PC2). Glucose AUC, mean glucose concentration, and HOMA-β contribute in similar fashions to both PCs. Thus, this method reduces eight distinct glucose homeostasis variables to two integrated response variables. For the anthropometric data, the first PC explained 54.2% of all variation, largely representing changes in estimates of fat mass; and the second PC (19.0% of the variation) represented responses in hip and waist measurements (Figure S2).

In order to understand whether the baseline glucose response cluster divided patients according to these integrated metrics created by principal components analysis (PCA), we assigned each patient PC1 and PC2 scores from the glucose homeostasis and anthropometric PCAs. PC scores are the values that individual patients take along particular PCs. For example, a patient with a specific set of glucose homeostasis parameter values would receive one specific PC1 score from the glucose homeostasis PCA, and this value would represent the value of that patient along the PC index, which explains most of the variation in the data. For glucose homeostasis, PC1 was dominated by insulin sensitivity indices, so this patient’s PC1 value would mostly, but not exclusively, represent changes in insulin sensitivity.

**Figure 3:**
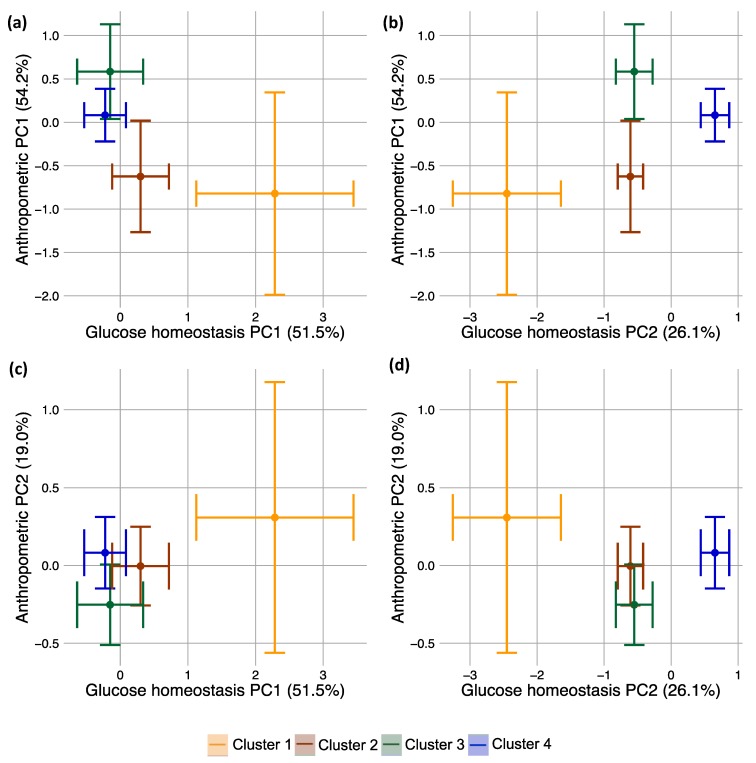
(**a**–**d**) Principal responses generated from principal components analysis of 1 year responses in individual parameters of both the glucose homeostasis and anthropometric parameter groups. PC stands for principal component. Points are model-adjusted means of each cluster from mixed models adjusting for sex; error bars are for standard errors. Percentages represent the amount of total variation in the data that is explained by a PC. (**a**,**b**) compare anthropometric PC1 against the glucose homeostasis PCs; (**c**,**d**) compare anthropometric PC2 against the glucose homeostasis PCs.

We then tested the power of baseline glucose response cluster to discriminate among patients’ PC scores (i.e., to discriminate among all integrated glucose homeostasis and anthropometric measurements created by PCA) (Figure 3, Table S7). Strikingly, baseline clusters were significantly different for glucose homeostasis PC2, indicating significant differences in the subjects’ integrated response, especially in HbA1c which is the individual parameter loading most strongly onto PC2. Indeed, Cluster 4 was significantly different from the others, forming a gradient of increasingly strong HbA1c improvement from Cluster 1 improving the least to Cluster 4 improving the most. Interestingly, Cluster 3 showed the greatest improvement in both anthropometric PC’s, whereas Cluster 4 showed the greatest improvement in glucose homeostasis.

### 3.8. Diabetes Remission

For patients with diabetes, a primary objective of bariatric surgery is diabetes remission, so we tested the predictive power of baseline glucose response clusters in a logistic framework, where a positive outcome was the absence of diabetes. The OGTT cluster alone provided little predictive power (mean AUC = 0.52 averaged across follow-up visits), but when combined with simple patient history questions like self-reported duration of diabetes, height, and weight, its mean AUC across follow-up times increases to 0.83 compared to mean AUC 0.69 from history variables alone. By including HbA1c, C reactive protein, and the number of medications, the model became a perfect predictor for diabetes remission (Figure S3, Table S8).

## 4. Discussion

Understanding heterogeneity in response to metabolic surgery is an outstanding goal in obesity and diabetes care. Defining improvement in multiple parameters simultaneously is complicated by the fact that parameters can change on very different scales. Often predicting multiple response parameters requires that multiple predictors be measured, a costly and time-intensive procedure [39,40]. To tackle this problem, we followed a data-driven approach to construct both a predictor (latent OGTT trajectories) and responses (PCs). For predictors, we used a latent trajectory algorithm to construct a dysglycemic index in patients. Based upon glucose load during OGTTs, latent trajectories uncovered four distinct clusters of patients that explained many short-term features of the surgery, especially glucose homeostasis. These clusters provided an intuitive surgical improvement index, ordering patients from the least dysglycemic and least improved to the most dysglycemic and most improved. Strikingly among glucose homeostasis parameters, insulin sensitivity was most altered under the bariatric surgery and was most improved in clusters with the greatest dysglycemia (cluster 4). To our knowledge, this was the first time that this method was simultaneously applied to patients both with and without diabetes.

A major goal of diabetes care is to establish a monitoring index that can integrate important information about a patient’s diabetic state on different scales. OGTTs capture dysregulations that occur on fine timescales since the entire test lasts only 2 h. These hour-scale measurements are likely relevant to the extremely rapid glycemic improvement that follows metabolic surgery, which may occur within hours of surgery and certainly occurs within days [14,41]. However, we also showed that clusters based upon glucose concentration during OGTTs were similarly able to discriminate among groups of longer-term glucose metrics like HbA1c (Table 1). HbA1c integrates the average glucose load to which hemoglobin is exposed over the lifetime of red blood cells, which is approximately three months. OGTTs, on the other hand, last only two hours, yet clusters based upon them established discrete classes of significantly increasing HbA1c from Cluster 1 to Cluster 4.

Interestingly, these clusters were remarkably discordant with dysglycemia diagnoses. In Western diabetes clinics, the majority of diagnoses rely upon HbA1c measurements [42]. Our results show that while OGTT clusters can predict relative HbA1c differences, diagnoses based upon standard criteria like HbA1c may obscure important diabetes differences. For example, applying the standard diabetes diagnosis criteria to this cohort identified 13 ostensibly normoglycemic patients at the baseline. However, of these patients, only two belonged to Cluster 1, the “healthiest” OGTT pattern (Table S2). This supports the increasingly skeptical view of current diabetes classifications that may be too coarse to differentiate among patient subpopulations that are pathophysiologically distinctive in glucose dysregulation [6]. Rather, these obese patients who appeared healthy from a diabetes perspective did, in fact, harbor latent dysglycemia that was detectable by closely analyzing OGTTs. Furthermore, two clusters occurred in patients with treated diabetes. This likely reflects differential responses among patients to drug therapy—those in Cluster 3 responding more healthily to an oral glucose challenge than the highly pathological response in Cluster 4. Together, these results point to the important potential role that latent trajectory algorithms could play as a useful tool in metabolic surgery clinics for understanding subtle patterns in patients’ responses to treatment that may be obscured if clinicians use only standard diagnostic criteria.

It is vital that clinicians be able to rapidly and intuitively interpret post-operative patient progress. For patients with diabetes, the goals of bariatric surgery include diabetes improvement and remission. Although including OGTT provided relatively modest improvement for predicting diabetes remission compared to patient history variables alone (increasing from 0.69 to 0.83, averaged across follow-up visits), these models were only used to predict the binary outcome of remission vs. no remission in the relatively small number of patients that were diabetic at the beginning of the study (30%). Rather, it may be more desirable to predict quantitative outcomes of bariatric surgery rather than binary events that are classified according to arbitrary criteria like remission guidelines. OGTT cluster proved extremely useful in explaining quantitative outcomes, improving the *R*^2^s of regression models by 28.8% averaged across all glucose homeostasis parameters. Thus, we have shown that including an OGTT cluster can help a clinician to predict quantitative responses in variables like HbA1c, as well as in the more limited cases of binary responses, like diabetes remission.

In most clinical settings, OGTTs are two-point tests at 0 and 120 min, and the utility of a multi-point version has been unclear [43]. However, we have shown that adding measurements at 30′ and 60′ enables the detection of cryptic differences in glucose decay patterns among patients not readily apparent at 0′ and 120′. In particular, latent trajectories built from these four-point tests form a discrete gradient of increasing dysglycemia from Cluster 1 to 4, with Cluster 1 being largely euglycemic, 1 and 2 peaking at 30 min, 3 and 4 peaking around 60 min, and 4 being the most dysglycemic. Furthermore, diabetes is a heterogenous disease with distinct etiologies. Some types emerge from a combination of obesity and insulin resistance while others emerge from a diminished beta-cell function, and these causes are not mutually exclusive [44]. A major goal in diabetes care has been to use a single test to intuitively differentiate subtypes and predict outcomes of specific interventions. Analyses of glucose load during OGTT have shown great promise in the former regard but not in the latter—OGTTs have not been used extensively to understand differences in responses to therapeutic interventions. For example, our results confirm previous findings based upon clusters of OGTT patterns that the most euglycemic cluster (Cluster 1) had the lowest triglyceride and total cholesterol levels [7,26]. Here, we extend the use of this clustering to a heterogenous population patients with and without diabetes to understand their responses to bariatric surgery.

Several limitations apply to this study. Notably, the sample size upon which oral glucose tolerance trajectories were calculated was not large (*n* = 154), consisting entirely of Polish Caucasians. We were not able to perform prospective validation of this method with an independent cohort, since our sample size was too small to cross-validate as training and validation subsets, and since there are very few datasets of multipoint point OGTTs for patients who have undergone bariatric surgery. This indicates that the utility of our latent trajectories may be limited to clinical settings similar to ours. Furthermore, there were relatively few patients with diabetes at baseline (30%), although a major point of our analysis was that diabetic criteria may obscure important pathological differences that differentiate patient subpopulations in a more pathophysiologically relevant way. Finally, only 35% of the cohort had OGTTs performed at every visit during the study period since many patients did not wish to stay for the approximately 2 h that the OGTTs required in addition to other measurements taken during this study. The impact of this missing data is mitigated by the majority of analyses being based upon baseline OGTT classification, which all analyzed patients had. The only inference relying upon longitudinal OGTT data was the qualitative result that the glucose cluster number decreased over time. While it is possible that 35% of patients with complete longitudinal data represent a non-random sample, it is extremely unlikely that this sample would, by chance, produce a false signal of a uniformly decreasing cluster number across time.

Thus, we propose that clusters of glucose load during OGTT may fulfill the need for a data-driven approach to predict the effectiveness of bariatric surgery on subpopulations of patients that is both pathophysiologically insightful and clinically tractable. OGTTs measure short-term dysglycemic states that are important for patients’ day-to-day lives, but we showed that OGTT clusters simultaneously explained longer term responses. These clusters revealed cryptic heterogeneity in dysglycemic pathologies of patients undergoing bariatric surgery and that their responses to bariatric surgery were correlated with the OGTT cluster. Although the physiological basis of these clusters remains unresolved, OGTT curves analyzed as latent trajectories may be an extremely effective metabolic screening tool to rapidly understand maximal clinically relevant information with minimal testing effort.

## 5. Conclusions

Latent trajectory analyses can be used to detect subtle dysglycemic features in obese patients using glucose load measurements taken during oral glucose tolerance tests (OGTTs). Clusters of these latent trajectories stratify patients into pathophysiologically insightful groups. These clusters help to explain outcomes of bariatric surgery in anthropometric and glucose homeostasis parameters, and improve prediction of diabetes remission.

## Figures and Tables

**Table 1:** Measurements gathered in this study divided into general metabolic, glucose homeostasis, and anthropometric parameter categories. Glucose and insulin concentrations were measured at 4 time points during the oral glucose tolerance test (0, 30, 60, and 120 min) and then used to calculate their area-under-the-curves. Principal components analysis was performed for variables found within these variable groups. HDL cholesterol, high-density lipoprotein cholesterol; LDL cholesterol, low-density lipoprotein cholesterol; OGTT, oral glucose tolerance test; BMI, body mass index.

General Metabolic	Glucose Homeostasis	Anthropometric
Total cholesterol	Glucose, OGTT	Waist-to-hip ratio
Triglycerides	Insulin, OGTT	Total body mass
HDL cholesterol	Glycated hemoglobin	Lean body mass
LDL cholesterol	HOMA-β	Visceral adipose tissue mass
Aspartate transaminase	HOMA-IR	Fat mass
Alanine transaminase	Matsuda index	BMI
C reactive protein		Weight

**Table 2:** Medians (interquartile range) at baseline divided by OGTT cluster. p-val is for p-value from non-parametric median tests testing whether medians significantly differed. NA, not applicable; HOMA, homeostasis model assessment; AUC, area under the curve; MET, metabolic equivalent of task; Bold indicates significant p-values (*p* < 0.05).

Variable Name	Cluster 1	Cluster 2	Cluster 3	Cluster 4	*p*-Value
Cohort size, *n* (%)	6 (4)	37 (27)	31 (22)	64 (46)	NA
Age, years	45 (39–47)	38 (31–48)	52 (43–54)	46 (39–57)	0.26
Male sex, *n* (%)	4 (66)	14 (38)	14 (45)	31 (48)	NA
Smoking, *n* (%)	0 (0)	4 (11)	7 (23)	9 (14)	NA
Total body mass, kg	146.2 (130.8–169.03)	128.6 ( 116.33–149.18)	133.1 (119.42–151.42)	138.4 (117.6–148.9)	0.39
Fat mass, kg	65.06 (64.55–76.23)	65.96 (54.75–75.79)	62.89 (53.38–70.98)	63.31 (53.8–70.45)	0.31
Lean body mass, kg	81.81 (64.73–88.95)	62.49 (53.05–67.9)	63.56 (55.37–76.76)	68.22 (57.15–76.64)	**0.03**
Visceral adipose tissue mass, kg	3.71 (2.5–4.98)	2.39 (1.98–3.55)	3.63 (2.41–4.29)	3.75 (2.8–4.99)	0.14
BMI, kg/m^2^	46.83 (45.45–51.58)	44.43 (40.65–49.69)	45.54 (41.82–50.39)	45.12 (42.37–49.44)	0.26
Fasting glucose, mmol/L	5.52 (5.31–6.10)	5.88 (5.66–6.04)	6.32 (5.93–6.90)	6.90 (6.36–7.99)	**<0.001**
Fasting insulin, pmol/L	244.45 (172.63–306.44)	138.04 (109.70–186.04)	210.72 (171.69–322.80)	247.89 (174.42–354.37)	**0.002**
HbA1c, %	5.20 (5.03–5.30)	5.50 (5.30–5.70)	5.90 (5.60-6.05)	6.15 (5.82–6.68)	**<0.001**
HbA1c, mmol/mol	33 (31–34)	37 (34–39)	41 (38–43)	44 (40–49)	**<0.001**
HOMA-beta	301.90 (187.09–562.72)	176.45 (122.13–269.72)	201.08 (142.22–309.90)	182.24 (125.88–292.69)	0.65
HOMA-IR	8.29 (6.76–10.30)	5.20 (3.73–6.76)	9.63 (6.06–12.76)	11.09 (7.61–16.17)	**<0.001**
Matsuda index	1.43 (1.22–1.78)	2.13 (1.64–2.48)	1.18 (0.9–1.58)	0.95 (0.72–1.48)	**<0.001**
Glucose AUC	231.12 (224.25–236.31)	291.50 (276.75–298.00)	338.00 (312.75–381.62)	418.75 (363.69–470.00)	**<0.001**
Insulin AUC	219.81 (209.13–277.50)	207.73 (159.35–271.18)	294.48 (180.43–392.15)	269.30 (183.53–364.25)	**0.03**
Mean insulin concentration during OGTT, pmol/L	711.18 (692.38–920.26)	614.89 (479.00–801.80)	883.67 (563.16–1145.77)	844.49 (588.70–1142.40)	**0.01**
Mean glucose concentration during OGTT, mmol/L	6.27 (6.11–6.54)	7.63 (7.27–7.92)	8.76 (8.11–9.83)	10.81 (9.44–12.11)	**<0.001**
Total cholesterol, mmol/L	4.18 (4.12–5.01)	5.10 (4.19–5.77)	5.20 (4.46–6.28)	4.79 (4.13–5.60)	0.37
Triglycerides, mmol/L	1.08 (0.83–1.37)	1.35 (0.98–1.78)	1.68 (1.27–2.00)	1.61 (1.2–2.54)	**0.02**
HDL-cholesterol, mmol/L	1.08 (0.95–1.2)	1.24 (1.01–1.45)	1.1 (0.91–1.45)	1.08 (0.95–1.37)	0.57
LDL-cholesterol, mmol/L	2.75 (2.64–2.95)	3.15 (2.69–3.87)	3.42 (2.73–4.00)	3.10 (2.38–3.63)	0.31
Aspartate transaminase, ukat/L	0.43 (0.34–0.45)	0.38 (0.29–0.45)	0.34 (0.29–0.42)	0.42 (0.32–0.56)	**0.04**
Alanine transaminase, ukat/L	0.54 (0.39–0.69)	0.47 (0.35–0.78)	0.47 (0.36–0.61)	0.56 (0.40–0.93)	0.56
C Reactive Protein, nmol/L	30.19 (15.04–32.47)	49.90 (30.19–75.99)	44.28 (26.76–81.61)	59.8 (33.23–107.23)	0.26
Physical activity, METs-minutes/week	3586 (3067–8899)	5937 (2046–12546)	5364 (2820–8773)	4513 (2355–13598)	0.71
Daily kcal intake, kcal/day	1309 (1079–1469)	1788 (1431–2209)	1578 (1284–2284)	1724 (1347–2334)	0.14

**Table 3:** The amount of variation in total time-series data explained by repeated measures mixed models including diagnosis, OGTT cluster, or both for the different response types. The parameters included for each response type are listed in Table 1. A separate model was fit for each response parameter, a marginal *R*^2^ calculated for each, and then averaged within response types. *N* is for the number of response variables within a response type to which a model was fit; μ*R*^2^ is mean marginal coefficient of multiple determination; lwr is the lower 95% confidence limit; upr is the upper 95% confidence limit.

Response Type	Model Terms	*N*	*μR* ^2^	lwr	upr
Anthropometric	diagnosis	12	0.483	0.39	0.57
	OGTT cluster	12	0.482	0.39	0.57
	diagnosis + OGTT cluster	12	0.492	0.40	0.58
Glucose homeostasis	diagnosis	11	0.319	0.22	0.49
	OGTT cluster	11	0.326	0.23	0.42
	diagnosis + OGTT cluster	11	0.411	0.30	0.52
General metabolic	diagnosis	11	0.185	0.12	0.25
	OGTT cluster	11	0.171	0.10	0.24
	diagnosis + OGTT cluster	11	0.208	0.14	0.28

## Supplementary Materials

The following are available online at https://www.mdpi.com/2077-0383/8/8/1091/s1, Table S1: Model selection for linear versus quadratic time series models for each parameter. Table S2: Two ways of classifying patients: diagnosis and OGTT cluster. Table S3: Quantile regression-estimated medians (IQRs) at baseline divided by OGTT cluster. Table S4: Generalized linear mixed models comparing time points for the entire patient cohort within each parameter controlling for age, sex, and smoking status. Table S5: Percent change (% change) from baseline to 12-month follow-up calculated from LS means ([final LS mean–baseline LS mean]/baseline LS mean) x 100 from time series models adjusting for age, sex, and smoking status. Table S6: Factor level tests on generalized linear mixed models comparing time points for OGTT-specific cross sections with predictors time + OGTT cluster + time × OGTT cluster, controlling for age, sex, and smoking status. Table S7: Post-hoc tests on generalized linear mixed models comparing principal component scores as “principal responses” to bariatric surgery among OGTT clusters controlling for age, sex, and smoking status. Table S8: Nested model performance predicting diabetes improvement and remission from baseline parameters. Figure S1: Biplot from principal components analysis of glucose homeostasis parameters (Table S1) with loadings plotted as arrows for each parameter and individual patients plotted as scores along PC1 and PC2. Figure S2: Biplot from principal components analysis of anthropometric parameters (Table S1) with loadings plotted as arrows for each parameter and individual patients plotted as scores along PC1 and PC2. Figure S3: Receiver operating characteristic curves from multinomial regression predicting diabetes remission.
