# Supplementary material for: Clusters of Glycemic Response to Oral Glucose Tolerance Tests Explain Multivariate Metabolic and Anthropometric Outcomes of Bariatric Surgery in Obese Patients
Source: J Clin Med. 2019 Jul 24;8(8):1091. doi: 10.3390/jcm8081091 (PMC6723855; doi:10.3390/jcm8081091)
Supplement: Supplementary file 1 [file jcm-08-01091-s001.pdf]

SUPPLEMENTAL MATERIAL

Clusters of glycemic response to oral glucose tolerance tests explain multivariate metabolic and anthropometric outcomes of bariatric surgery in obese patients

Lukasz Szczerbinski MD, PhD, Mark A. Taylor PhD, Anna Citko Msc, Maria Gorska MD, PhD, Steen Larsen PhD, Hady Razak Hady MD, PhD, Adam Kretowski MD, PhD

Author for correspondence: Lukasz Szczerbinski
Email: lukasz.szczerbinski@umb.edu.pl

This file includes:

Figures S1 to S3
Tables S1 to S7

parameter	model	AIC	BIC	χ^2	p
Adiponectin	linear	4587	4628	7.0	0.13360
	quadratic	4588	4642		
Alanine transaminase	linear	5449	5502	12.8	0.01246
	quadratic	5445	5515		
Aspartate transaminase	linear	4617	4669	13.7	0.00831
	quadratic	4611	4681		
BMI	linear	3396	3449	361.1	0.00000
	quadratic	3043	3113		
C Reactive Protein	linear	3705	3758	12.4	0.01483
	quadratic	3701	3771		
Fat mass	linear	12556	12608	186.0	0.00000
	quadratic	12378	12448		
Glucose AUC	linear	4208	4255	105.3	0.00000
	quadratic	4111	4174		
HbA1c	linear	744	796	106.9	0.00000
	quadratic	645	715		
HDL cholesterol	linear	4435	4487	32.4	0.00000
	quadratic	4410	4480		
Hip circumference	linear	4331	4383	142.8	0.00000
	quadratic	4196	4266		
HOMA-beta	linear	6967	7020	48.0	0.00000
	quadratic	6927	6998		
HOMA-IR	linear	3362	3415	145.2	0.00000
	quadratic	3225	3295		
Insulin AUC	linear	4645	4692	18.7	0.00091
	quadratic	4634	4697		
LDL cholesterol	linear	5591	5643	1.9	0.75037
	quadratic	5597	5667		
Lean body mass	linear	11723	11776	179.3	0.00000
	quadratic	11552	11622		
Matsuda index	linear	2431	2481	35.1	0.00000
	quadratic	2404	2470		
Mean glucose concentration during OGTT	linear	5867	5920	94.9	0.00000
	quadratic	5780	5850		
Mean insulin concentration during OGTT	linear	6549	6602	31.1	0.00000
	quadratic	6526	6596		
Total body mass	linear	4691	4744	327.4	0.00000
	quadratic	4372	4442		
Total cholesterol	linear	5838	5891	15.4	0.00392
	quadratic	5831	5901		
Triglycerides	linear	6530	6583	44.8	0.00000
	quadratic	6493	6564		
Visceral adipose tissue mass	linear	8496	8547	151.6	0.00000
	quadratic	8352	8420		
Waist circumference	linear	4452	4504	189.2	0.00000

	quadratic	4270	4341		
Waist to hip ratio	linear	-1766	-1714	16.3	0.00263
	quadratic	-1775	-1705		

Table S1. Model selection for linear versus quadratic time series models for each parameter. AIC refers to the Akaike Information Criterion, and BIC refers to the Bayesian Information Criterion calculated for each time series model. χ^2 reports the test statistic value on the likelihood ratio test performed between the linear and quadratic models for a particular parameter, and p refers to the p-value for that test. There were 4 degrees of freedom for all tests. Significant p-values ($p < 0.05$) indicates significant differences between models and lower information criteria indicate more informative models.

	Cluster 1	Cluster 2	Cluster 3	Cluster 4
**Non-diabetic**	2	9	2	0
**IFG**	3	22	21	8
**IFG + IGT**	0	5	1	20
**untreated T2D**	0	0	4	16
**treated T2D**	0	0	2	19

Table S2. Two ways of classifying patients: diagnosis and OGTT cluster. Individually, OGTT cluster and diagnosis totals do not match with the intersecting totals between cluster and diagnosis because baseline diagnosis was missing from 4 of 138 analyzed patients (i.e., they underwent bariatric surgery before a definitive diabetes diagnosis could be established).

Variable name	Model	Class 1	Class 2	Class 3	Class 4
Total body mass, kg	1	NA	126.29 (114.04-136.73)	130.25 (118.83-152.13)	129.3 (119.4-141.15)
	2	NA	129.89 (117.45-138.38)	129.23 (116.01-152.84)	128.78 (116.76-141.53)
Fat mass, kg	1	NA	63.17 (59.12-69.81)	61.81 (56.18-65.9)	63.34 (59.99-66.23)
	2	NA	65.59 (59.79-70.26)	60.34 (56.12-65.81)	62.51 (59.21-66.23)
Lean body mass, kg	1	NA	60.05 (54.69-72.23)	60.28 (55.74-78.79)	58.44 (54.36-77.29)
	2	NA	58.61 (54.16-72.73)	59.58 (54.53-79.36)	58.82 (55.3-77.02)
Visceral adipose tissue mass, kg	1	NA	2.09 (2.08-4.81)	3.24 (2.79-4.88)	3.29 (2.94-5.22)
	2	NA	2.12 (1.93-4.75)	3.22 (2.76-4.91)	3.31 (2.96-5.21)
BMI, kg/m^2	1	NA	44.48 (43.82-47.34)	43.67 (42.69-47.87)	45.35 (44.6-46.69)
	2	NA	44.48 (43.82-47.28)	44.54 (42.75-47.53)	45.15 (44.42-46.44)
Fasting glucose, momol/L	1	NA	5.88 (5.74-5.94)	6.36 (6.14-6.68)	7.04 (6.74-7.26)
	3	NA	5.79 (5.73-5.93)	6.34 (6.13-6.52)	7.09 (6.79-7.27)
Fasting insulin, pmol/L	1	NA	130.44 (115.73-156.70)	185.9 (170.40-312.04)	236.05 (168.89-357.38)
	3	NA	137.18 (119.60-171.48)	197.74 (157.85-290.30)	258.94 (184.82-375.75)
HbA1c, %	1	NA	5.50 (5.43-5.54)	5.80 (5.71-5.90)	6.15 (6.10-6.20)
	3	NA	5.50 (5.47-5.51)	5.87 (5.75-6.00)	6.20 (6.03-6.36)
HbA1c, mmol/mol	1	NA	37 (36-37)	40 (39-41)	44 (43-44)
	3	NA	37 (36-37)	41 (39-42)	44 (42-46)
HOMA-beta	1	NA	184.15 (155.14-241.11)	195.40 (161.77-301.94)	190.57 (133.53-251.61)
	3	NA	177.93 (135.89-236.46)	236.20 (157.01-311.66)	183.68 (137.55-238.32)
HOMA-IR	1	NA	4.83 (4.61-5.60)	7.14 (6.07-10.90)	11.32 (8.61-14.68)
	3	NA	4.64 (4.25-5.87)	6.78 (5.61-10.98)	11.41 (8.85-14.44)
Matsuda index	1	NA	2.13 (1.87-2.29)	1.55 (0.98-1.55)	1.07 (0.76-1.25)
	3	NA	2.22 (1.78-2.48)	1.50 (1.00-1.59)	0.97 (0.72-1.23)
Glucose AUC	1	NA	289.02 (280.56-293.60)	353.82 (333.87-359.72)	425 (399.56-439.44)
	3	NA	288.25 (281.76-292.21)	349.04 (340.08-363.67)	422.88 (390.57-440.9)
Insulin AUC	1	NA	201.24 (185.52-229.36)	230.70 (215.44-365.46)	249.90 (235.00-273.94)
	3	NA	193.72 (168.78-240.87)	253.16 (191.81-385.79)	273.54 (233.49-303.45)
Mean insulin concentration during OGTT, pmol/L	1	NA	612.52 (551.25-722.09)	630.82 (615.47-1144.69)	779.85 (726.46-873.62)
	3	NA	637.57 (544.43-714.91)	740.89 (572.7-1149.14)	825.91 (730.27-929.44)
Mean glucose concentration during OGTT, mmol/L	1	NA	7.57 (7.39-7.72)	9.01 (8.53-9.24)	10.78 (10.14-11.27)
	3	NA	7.59 (7.39-7.89)	8.98 (8.68-9.29)	10.79 (10-11.27)
Total cholesterol, mmol/L	1	NA	5.08 (4.37-5.27)	5.16 (5.08-5.26)	4.60 (4.53-5.00)
	3	NA	4.92 (4.43-5.61)	5.32 (4.76-5.81)	4.87 (4.45-5.28)
Triglycerides, mmol/L	1	NA	1.45 (1.19-1.58)	1.54 (1.41-1.83)	1.60 (1.55-1.69)
	3	NA	1.35 (1.16-1.50)	1.69 (1.44-1.86)	1.71 (1.51-1.84)
HDL-cholesterol, mmol/L	1	NA	1.37 (0.93-1.37)	1.16 (1.01-1.25)	1.13 (1.02-1.20)
	3	NA	1.29 (1.09-1.42)	1.12 (1.00-1.33)	1.12 (1.03-1.18)

**LDL-cholesterol, mmol/L**	1	NA	3.15 (2.79-3.35)	3.55 (3.43-3.75)	2.97 (2.93-3.35)
	3	NA	3.17 (3.03-3.43)	3.23 (2.84-3.72)	2.93 (2.77-3.43)
**Aspartate transaminase, ukat/L**	1	NA	0.31 (0.30-0.44)	0.35 (0.33-0.36)	0.39 (0.32-0.50)
	3	NA	0.31 (0.29-0.42)	0.36 (0.34-0.37)	0.42 (0.36-0.50)
**Alanine transaminase, ukat/L**	1	NA	0.41 (0.33-0.70)	0.45 (0.38-0.64)	0.58 (0.42-0.91)
	3	NA	0.39 (0.36-0.71)	0.48 (0.41-0.59)	0.64 (0.49-0.80)
**C Reactive Protein, nmol/L**	1	NA	57.80 (47.33-70.47)	50.76 (46.19-63.42)	61.99 (57.33-69.61)
	3	NA	52.57 (35.23-75.23)	50.38 (39.42-55.14)	55.99 (45.52-72.85)
**Physical activity, METs-minutes/week**	1	NA	6233 (5624-6484)	5388 (5050-6664)	5214 (3131-6851)
	3	NA	5514 (3550-7669)	4854 (3580-6491)	4698 (2985-7218)
**Daily kcal intake, kcal/day**	1	NA	1714 (1702-2093)	1640 (1472-2210)	1724 (1493-1966)
	2	NA	1755 (1718-2145)	1679 (1412-2108)	1724 (1517-1966)

Table S3. Quantile regression-estimated medians (IQRs) at baseline divided by OGTT cluster. Model 1 controlled for age and sex; model 2 controlled for age, sex, and smoking status; and model 3 for age, sex, smoking status, and baseline BMI. NA indicated unstable model estimates in cluster 1 because of insufficient sample size (n = 6).

parameter	time	mean	SE	df	LCL	UCL	CLD
**Waist circumference (cm)**	baseline	136.83	1.32	137	134.23	139.44	5
	1 mo	126.64	1.32	137	124.03	129.24	4
	3 mo	119.2	1.32	137	116.58	121.82	3
	6 mo	110.56	1.34	137	107.91	113.21	2
	12 mo	105.16	1.41	137	102.39	107.94	1
**Hip circumference (cm)**	baseline	135.24	1.2	137	132.86	137.62	5
	1 mo	128.07	1.2	137	125.69	130.45	4
	3 mo	121.5	1.21	137	119.1	123.89	3
	6 mo	115.38	1.23	137	112.96	117.81	2
	12 mo	111.52	1.29	137	108.97	114.07	1
**Waist to hip ratio**	baseline	1.02	0.01	137	1	1.03	3
	1 mo	0.99	0.01	137	0.98	1.01	2
	3 mo	0.98	0.01	137	0.97	1	2
	6 mo	0.96	0.01	137	0.94	0.97	1
	12 mo	0.94	0.01	137	0.93	0.96	1
**Total body mass (kg)**	baseline	135.74	2.02	136	131.74	139.73	5
	1 mo	122.01	2.02	136	118.02	126.01	4
	3 mo	111.5	2.02	136	107.5	115.5	3
	6 mo	103.41	2.03	136	99.39	107.44	2
	12 mo	98.93	2.08	136	94.82	103.04	1
**Fat mass (kg)**	baseline	65.81	1.32	136	63.20	68.42	5
	1 mo	58.36	1.32	136	55.75	60.96	4
	3 mo	48.38	1.32	136	45.76	50.99	3
	6 mo	41.34	1.34	136	38.69	43.99	2
	12 mo	36.26	1.40	136	33.49	39.03	1
**Lean body mass (kg)**	baseline	66.80	1.06	136	64.70	68.90	3
	1 mo	60.45	1.06	136	58.36	62.55	2
	3 mo	60.02	1.06	136	57.92	62.12	12
	6 mo	59.73	1.07	136	57.62	61.84	12
	12 mo	59.47	1.08	136	57.33	61.60	1
**BMI (kg/m^2)**	baseline	46.3	0.54	137	45.23	47.37	5
	1 mo	41.65	0.54	137	40.58	42.72	4

	3 mo	38.1	0.54	137	37.03	39.18	3
	6 mo	35.29	0.55	137	34.21	36.37	2
	12 mo	33.73	0.56	137	32.62	34.85	1
**Physical activity (METs-minutes/week)**	baseline	9047	839	137	7389	10706	12
	1 mo	6675	851	137	4991	8358	1
	3 mo	11177	857	137	9483	12871	23
	6 mo	10835	904	137	9046	12623	23
	12 mo	13791	1052	137	11711	15871	3
**HbA1c (%)**	baseline	5.95	0.05	137	5.86	6.05	3
	1 mo	5.55	0.05	137	5.46	5.64	2
	3 mo	5.38	0.05	137	5.28	5.47	1
	6 mo	5.36	0.05	137	5.27	5.46	1
	12 mo	5.27	0.05	137	5.16	5.38	1
**HOMA-beta**	baseline	230.53	9.05	137	212.64	248.43	4
	1 mo	183.2	9.08	137	165.24	201.15	3
	3 mo	157.43	9.16	137	139.31	175.56	2
	6 mo	138.99	9.46	137	120.28	157.7	12
	12 mo	129.16	10.56	137	108.28	150.03	1
**HOMA-IR**	baseline	10.32	0.36	137	9.62	11.03	3
	1 mo	6.06	0.36	137	5.36	6.77	2
	3 mo	4.29	0.36	137	3.58	5.01	1
	6 mo	3.47	0.38	137	2.72	4.22	1
	12 mo	3.3	0.44	137	2.43	4.17	1
**Mean insulin concentration during OGTT (pmol/L)**	baseline	866.96	30.85	137	806.04	927.94	4
	1 mo	155.48	31.00	137	94.21	216.69	1
	3 mo	554.56	31.35	137	492.56	616.55	3
	6 mo	489.55	32.79	137	424.69	554.48	23
	12 mo	416.44	37.81	137	341.67	491.20	2
**Mean glucose concentration during OGTT (mmol/l)**	baseline	9.47	0.14	137	9.19	9.74	4
	1 mo	6.05	0.14	137	5.78	6.32	1
	3 mo	7.17	0.14	137	6.90	7.45	3
	6 mo	6.98	0.15	137	6.69	7.26	23
	12 mo	6.68	0.17	137	6.35	7.01	2
**Matsuda index**	baseline	1.48	0.31	137	0.87	2.1	1

	3 mo	4.18	0.32	137	3.55	4.81	2
	6 mo	5.01	0.33	137	4.35	5.67	23
	12 mo	5.69	0.39	137	4.92	6.45	3
**Glucose AUC**	baseline	364.41	6.37	137	351.81	377.01	2
	3 mo	288.05	7	137	274.21	301.9	1
	6 mo	280.27	7.29	137	265.86	294.68	1
	12 mo	278.47	8.57	137	261.52	295.43	1
**Insulin AUC**	baseline	280.05	10.99	136	258.32	301.78	2
	3 mo	217	12.08	136	193.1	240.9	1
	6 mo	201.96	12.67	136	176.89	227.02	1
	12 mo	186.8	14.87	136	157.39	216.21	1
**Total cholesterol (mmol/l)**	baseline	5.01	0.08	137	4.85	5.17	3
	1 mo	4.45	0.08	137	4.29	4.61	1
	3 mo	4.63	0.08	137	4.47	4.80	12
	6 mo	4.81	0.09	137	4.64	4.98	23
	12 mo	4.82	0.10	137	4.64	5.01	23
**Triglycerides (mmol/l)**	baseline	1.80	0.06	137	1.68	1.91	3
	1 mo	1.52	0.06	137	1.41	1.64	2
	3 mo	1.23	0.06	137	1.12	1.35	1
	6 mo	1.19	0.06	137	1.07	1.31	1
	12 mo	1.14	0.07	137	1.00	1.28	1
**HDL cholesterol (mmol/l)**	baseline	1.19	0.03	136	1.14	1.25	2
	1 mo	0.98	0.03	136	0.93	1.03	1
	3 mo	1.16	0.03	136	1.11	1.21	2
	6 mo	1.33	0.03	136	1.28	1.39	3
	12 mo	1.52	0.03	136	1.47	1.58	4
**LDL cholesterol (mmol/l)**	baseline	3.15	0.07	137	3.02	3.29	2
	1 mo	2.84	0.07	137	2.70	2.97	1
	3 mo	3.04	0.07	137	2.90	3.17	2
	6 mo	3.11	0.07	137	2.97	3.25	2
	12 mo	3.08	0.08	137	2.92	3.24	2
**Aspartate transaminase (ukat/l)**	baseline	0.47	0.02	137	0.43	0.50	2
	1 mo	0.48	0.02	137	0.45	0.52	2
	3 mo	0.37	0.02	137	0.34	0.41	1

	6 mo	0.33	0.02	137	0.29	0.37	1
	12 mo	0.33	0.02	137	0.29	0.37	1
**Alanine transaminase (ukat/l)**	baseline	0.62	0.03	137	0.56	0.68	3
	1 mo	0.71	0.03	137	0.65	0.78	3
	3 mo	0.45	0.03	137	0.39	0.52	2
	6 mo	0.34	0.04	137	0.27	0.41	1
	12 mo	0.31	0.04	137	0.23	0.40	1
**C-Reactive Protein (nmol/l)**	baseline	64.29	4.57	137	55.33	73.24	3
	1 mo	46.19	4.48	137	37.24	55.05	2
	3 mo	42.10	4.57	137	33.05	51.14	12
	6 mo	30.00	4.86	137	20.38	39.52	1
	12 mo	29.71	5.71	137	18.38	40.95	12

Table S4. Generalized linear mixed models comparing time points for the entire patient cohort within each parameter controlling for age, sex, and smoking status. Mean refers to model-adjusted mean; SE for standard error; df for degrees of freedom; LCL for the lower 95% confidence limit; UCL for the upper 95% confidence limit; CLD for compact letter display. Times with different CLDs within a particular parameter indicates that those times are significantly different from each other.

variable	category	% change
Waist circumference	Anthropometric	23.1
Hip circumference	Anthropometric	17.5
Waist to hip ratio	Anthropometric	7.2
Total body mass	Anthropometric	27.1
Fat mass	Anthropometric	44.9
Lean body mass	Anthropometric	11.0
Visceral adipose tissue mass	Anthropometric	56.0
BMI	Anthropometric	27.2
Physical activity	exercise	52.4
HbA1c	Glucose homeostasis	11.4
HOMA-beta	Glucose homeostasis	44.0
HOMA-IR	Glucose homeostasis	68.0
Mean insulin concentration during OGTT	Glucose homeostasis	52.0
Mean glucose concentration during OGTT	Glucose homeostasis	29.4
Matsuda index	Glucose homeostasis	283.4
Glucose AUC	Glucose homeostasis	23.6
Insulin AUC	Glucose homeostasis	33.3
Total cholesterol	General metabolic	3.7
Triglycerides	General metabolic	36.4
HDL cholesterol	General metabolic	27.5
LDL cholesterol	General metabolic	2.3
Aspartate transaminase	General metabolic	29.0
Alanine transaminase	General metabolic	49.3
C Reactive Protein	General metabolic	53.8

Table S5. Percent change (% change) from baseline to 12-month follow-up calculated from LS means ([final LS mean – baseline LS mean] / baseline LS mean) x 100 from time series models adjusting for age, sex, and smoking status.

name	time	OGTT	mean	SE	df	LCL	UCL	CLD
Glycated hemoglobin	baseline	1	5.15	0.20	137	4.76	5.54	1234
	1 mo	1	4.88	0.20	137	4.49	5.28	12
	3 mo	1	4.95	0.20	137	4.56	5.34	12
	6 mo	1	5.13	0.20	137	4.74	5.53	1234
	12 mo	1	4.93	0.25	137	4.44	5.41	123
	baseline	2	5.47	0.08	134	5.31	5.63	234
	1 mo	2	5.19	0.08	134	5.03	5.35	1
	3 mo	2	5.15	0.08	134	4.99	5.31	1
	6 mo	2	5.17	0.09	134	5.00	5.34	1
	12 mo	2	5.09	0.10	134	4.89	5.28	1
	baseline	3	5.89	0.09	134	5.72	6.06	4
	1 mo	3	5.53	0.09	134	5.36	5.70	123
	3 mo	3	5.49	0.09	134	5.32	5.67	123
	6 mo	3	5.46	0.09	134	5.27	5.65	123
	12 mo	3	5.27	0.11	134	5.06	5.48	12
	baseline	4	6.35	0.06	134	6.23	6.47	5
	1 mo	4	5.83	0.06	134	5.71	5.95	34
	3 mo	4	5.50	0.06	134	5.37	5.62	12
	6 mo	4	5.46	0.06	134	5.33	5.58	12
	12 mo	4	5.43	0.07	134	5.29	5.56	12
HOMA-beta	baseline	1	406.33	42.78	137	321.74	490.91	6
	1 mo	1	231.45	44.80	137	142.87	320.04	12345
	3 mo	1	178.71	42.78	137	94.13	263.30	12345
	6 mo	1	188.71	42.78	137	104.12	273.30	12345
	12 mo	1	146.30	47.54	137	52.30	240.31	12345
	baseline	2	200.86	17.23	134	166.80	234.93	2345
	1 mo	2	181.24	17.23	134	147.17	215.31	12345
	3 mo	2	146.80	17.46	134	112.28	181.33	124
	6 mo	2	123.84	18.33	134	87.58	160.09	1
	12 mo	2	125.46	20.69	134	84.53	166.39	1
	baseline	3	239.00	18.82	134	201.78	276.22	456
	1 mo	3	179.26	18.96	134	141.76	216.77	123
	3 mo	3	145.52	19.47	134	107.02	184.03	123
	6 mo	3	122.46	20.10	134	82.71	162.21	12
	12 mo	3	110.97	23.06	134	65.35	156.58	12
	baseline	4	226.97	13.15	134	200.97	252.97	35
	1 mo	4	181.36	13.15	134	155.36	207.36	124
	3 mo	4	167.11	13.19	134	141.02	193.21	124
	6 mo	4	150.27	13.53	134	123.50	177.03	12
	12 mo	4	138.01	14.81	134	108.71	167.31	12
HOMA-IR	baseline	1	9.42	1.59	137	6.27	12.57	4567
	1 mo	1	5.29	1.59	137	2.14	8.44	123456
	3 mo	1	3.56	1.59	137	0.41	6.70	123456
	6 mo	1	3.71	1.59	137	0.56	6.86	123456
	12 mo	1	2.40	1.85	137	-1.25	6.06	123
	baseline	2	5.87	0.64	134	4.60	7.14	2345
	1 mo	2	4.86	0.64	134	3.59	6.13	12345
	3 mo	2	3.15	0.65	134	1.85	4.44	1
	6 mo	2	2.78	0.70	134	1.39	4.16	1
	12 mo	2	2.89	0.83	134	1.25	4.53	12

	baseline	3	9.68	0.70	134	8.29	11.06	6
	1 mo	3	6.03	0.71	134	4.63	7.43	12345
	3 mo	3	4.01	0.74	134	2.55	5.47	12345
	6 mo	3	3.30	0.77	134	1.78	4.82	124
	12 mo	3	3.00	0.93	134	1.16	4.83	124
	baseline	4	13.34	0.49	134	12.37	14.31	7
	1 mo	4	6.85	0.49	134	5.88	7.82	356
	3 mo	4	5.16	0.49	134	4.19	6.14	12345
	6 mo	4	3.97	0.51	134	2.96	4.98	124
	12 mo	4	3.86	0.58	134	2.71	5.01	124
Total cholesterol	baseline	1	180.83	15.02	137	151.13	210.54	12345
	1 mo	1	173.83	15.02	137	144.13	203.54	12345
	3 mo	1	171.17	15.02	137	141.46	200.87	12345
	6 mo	1	180.17	15.02	137	150.46	209.87	12345
	12 mo	1	176.53	16.73	137	143.45	209.61	12345
	baseline	2	193.24	6.05	134	181.28	205.21	345
	1 mo	2	163.00	6.05	134	151.04	174.96	12
	3 mo	2	174.62	6.13	134	162.49	186.75	1234
	6 mo	2	182.02	6.45	134	169.26	194.79	12345
	12 mo	2	183.04	7.31	134	168.57	197.50	12345
	baseline	3	208.94	6.61	134	195.87	222.01	5
	1 mo	3	188.85	6.66	134	175.68	202.03	12345
	3 mo	3	194.75	6.72	134	181.46	208.04	12345
	6 mo	3	199.92	7.07	134	185.92	213.91	45
	12 mo	3	196.00	7.98	134	180.22	211.79	12345
	baseline	4	187.22	4.60	134	178.12	196.31	245
	1 mo	4	168.62	4.62	134	159.49	177.75	13
	3 mo	4	174.42	4.63	134	165.26	183.59	1234
	6 mo	4	181.37	4.74	134	172.00	190.74	12345
	12 mo	4	183.98	5.19	134	173.72	194.24	12345
Triglycerides	baseline	1	97.33	23.85	137	50.16	144.50	12345678
	1 mo	1	119.50	23.85	137	72.33	166.67	12345678
	3 mo	1	108.00	23.85	137	60.83	155.17	12345678
	6 mo	1	100.67	23.85	137	53.50	147.84	12345678
	12 mo	1	111.65	27.66	137	56.95	166.36	12345678
	baseline	2	133.27	9.61	134	114.27	152.27	345678
	1 mo	2	111.30	9.61	134	92.30	130.30	12345
	3 mo	2	90.23	9.80	134	70.85	109.61	1
	6 mo	2	91.67	10.50	134	70.89	112.45	12
	12 mo	2	84.07	12.38	134	59.58	108.55	1
	baseline	3	175.58	10.49	134	154.82	196.34	78
	1 mo	3	159.77	10.61	134	138.78	180.77	5678
	3 mo	3	131.10	10.75	134	109.85	152.36	123456
	6 mo	3	123.66	11.53	134	100.85	146.47	123456
	12 mo	3	106.70	13.50	134	79.99	133.40	1234
	baseline	4	171.34	7.30	134	156.90	185.79	68
	1 mo	4	137.62	7.34	134	123.10	152.15	2457
	3 mo	4	109.34	7.38	134	94.73	123.94	1234
	6 mo	4	105.27	7.61	134	90.21	120.33	13
	12 mo	4	105.86	8.61	134	88.83	122.89	1234
HDL cholesterol	baseline	1	44.17	4.74	136	34.79	53.55	1234567890ABCDE

	1 mo	1	35.50	4.74	136	26.12	44.88	123456
	3 mo	1	41.67	4.74	136	32.29	51.05	1234567890ABCDE
	6 mo	1	50.83	4.74	136	41.45	60.21	7890ABCDEF
	12 mo	1	48.60	5.21	136	38.29	58.91	1234567890ABCDEF
	baseline	2	48.62	1.91	133	44.84	52.40	4560ABCDE
	1 mo	2	39.19	1.91	133	35.41	42.97	123789
	3 mo	2	46.19	1.93	133	42.37	50.02	4560AB
	6 mo	2	52.26	2.02	133	48.26	56.26	56ABCDE
	12 mo	2	63.35	2.26	133	58.88	67.83	F
	baseline	3	47.33	2.12	133	43.14	51.53	3569AB
	1 mo	3	39.89	2.14	133	35.66	44.11	124780
	3 mo	3	44.16	2.15	133	39.90	48.42	1234567890AB
	6 mo	3	49.89	2.23	133	45.47	54.30	56ABC
	12 mo	3	58.64	2.48	133	53.72	63.55	DEF
	baseline	4	44.30	1.45	133	41.42	47.17	2345890A
	1 mo	4	36.16	1.46	133	33.27	39.06	17
	3 mo	4	44.29	1.46	133	41.40	47.18	2345890A
	6 mo	4	51.67	1.49	133	48.72	54.62	6BD
	12 mo	4	57.56	1.61	133	54.36	60.75	CEF
LDL cholesterol	baseline	1	117.58	12.55	137	92.76	142.41	123
	1 mo	1	112.90	12.55	137	88.08	137.72	123
	3 mo	1	115.37	12.55	137	90.54	140.19	123
	6 mo	1	114.37	12.55	137	89.54	139.19	123
	12 mo	1	109.30	13.97	137	81.68	136.93	123
	baseline	2	122.54	5.06	134	112.54	132.54	23
	1 mo	2	103.48	5.06	134	93.48	113.47	1
	3 mo	2	114.02	5.13	134	103.88	124.16	123
	6 mo	2	118.86	5.39	134	108.20	129.53	123
	12 mo	2	112.33	6.11	134	100.25	124.40	123
	baseline	3	131.87	5.52	134	120.95	142.80	3
	1 mo	3	120.78	5.57	134	109.77	131.79	123
	3 mo	3	126.86	5.62	134	115.75	137.97	123
	6 mo	3	131.43	5.91	134	119.74	143.12	123
	12 mo	3	127.01	6.66	134	113.84	140.19	123
	baseline	4	116.92	3.84	134	109.31	124.52	123
	1 mo	4	107.29	3.86	134	99.66	114.92	12
	3 mo	4	114.59	3.87	134	106.93	122.25	123
	6 mo	4	116.21	3.96	134	108.38	124.04	123
	12 mo	4	119.25	4.33	134	110.69	127.82	123
Aspartate transaminase	baseline	1	23.27	4.93	137	13.53	33.01	1234
	1 mo	1	26.47	4.93	137	16.73	36.21	1234
	3 mo	1	21.02	4.93	137	11.28	30.76	1234
	6 mo	1	20.65	4.93	137	10.91	30.39	1234
	12 mo	1	24.12	5.77	137	12.70	35.53	1234
	baseline	2	28.32	1.98	134	24.40	32.24	234
	1 mo	2	28.43	2.00	134	24.47	32.40	234
	3 mo	2	21.99	2.03	134	17.99	26.00	1234
	6 mo	2	19.57	2.18	134	15.25	23.89	1
	12 mo	2	19.74	2.60	134	14.60	24.87	123
	baseline	3	23.64	2.17	134	19.36	27.93	1234
	1 mo	3	27.11	2.19	134	22.77	31.45	1234

	3 mo	3	21.20	2.25	134	16.74	25.66	1234
	6 mo	3	19.40	2.40	134	14.66	24.15	12
	12 mo	3	21.04	2.83	134	15.44	26.64	1234
	baseline	4	30.69	1.51	134	27.71	33.67	4
	1 mo	4	30.52	1.52	134	27.52	33.52	34
	3 mo	4	23.65	1.53	134	20.63	26.67	12
	6 mo	4	20.33	1.58	134	17.21	23.45	12
	12 mo	4	19.45	1.80	134	15.89	23.01	12
Alanine transaminase	baseline	1	31.95	9.43	137	13.30	50.60	123
	1 mo	1	40.63	9.43	137	21.99	59.28	123
	3 mo	1	24.67	9.43	137	6.02	43.31	123
	6 mo	1	21.77	9.43	137	3.12	40.41	123
	12 mo	1	21.79	11.18	137	-0.33	43.90	123
	baseline	2	33.79	3.80	134	26.28	41.31	123
	1 mo	2	47.41	3.80	134	39.90	54.92	3
	3 mo	2	26.27	3.89	134	18.58	33.95	12
	6 mo	2	21.07	4.21	134	12.74	29.40	1
	12 mo	2	17.63	5.06	134	7.62	27.64	1
	baseline	3	30.90	4.15	134	22.70	39.11	123
	1 mo	3	37.01	4.20	134	28.69	45.32	123
	3 mo	3	30.58	4.26	134	22.15	39.02	123
	6 mo	3	19.71	4.62	134	10.56	28.85	1
	12 mo	3	19.52	5.52	134	8.61	30.44	1
	baseline	4	43.11	2.89	134	37.40	48.82	23
	1 mo	4	43.19	2.91	134	37.45	48.94	23
	3 mo	4	26.80	2.93	134	21.02	32.59	1
	6 mo	4	20.05	3.03	134	14.06	26.05	1
	12 mo	4	19.26	3.49	134	12.36	26.15	1
C Reactive Protein	baseline	1	3.27	2.25	137	-1.18	7.72	12
	1 mo	1	3.44	2.25	137	-1.01	7.89	12
	3 mo	1	2.57	2.25	137	-1.88	7.02	12
	6 mo	1	2.36	2.25	137	-2.09	6.81	12
	12 mo	1	0.98	2.69	137	-4.34	6.29	12
	baseline	2	6.01	0.93	134	4.17	7.84	12
	1 mo	2	4.56	0.91	134	2.77	6.36	12
	3 mo	2	3.88	0.93	134	2.04	5.71	1
	6 mo	2	2.98	1.01	134	0.98	4.97	1
	12 mo	2	3.58	1.22	134	1.17	6.00	12
	baseline	3	5.60	0.99	134	3.64	7.55	12
	1 mo	3	3.92	1.00	134	1.94	5.91	12
	3 mo	3	4.24	1.02	134	2.23	6.26	12
	6 mo	3	3.04	1.11	134	0.84	5.23	1
	12 mo	3	4.93	1.33	134	2.30	7.56	12
	baseline	4	8.09	0.70	134	6.71	9.47	2
	1 mo	4	5.59	0.69	134	4.21	6.96	12
	3 mo	4	4.99	0.70	134	3.61	6.38	1
	6 mo	4	3.38	0.72	134	1.95	4.82	1
	12 mo	4	2.46	0.84	134	0.80	4.12	1
Waist to hip ratio	baseline	1	1.02	0.03	137	0.96	1.09	123456789
	1 mo	1	0.99	0.03	137	0.92	1.05	123456789
	3 mo	1	1.01	0.03	137	0.95	1.08	123456789

	6 mo	1	0.96	0.03	137	0.90	1.03	123456789
	12 mo	1	0.98	0.04	137	0.91	1.05	123456789
	baseline	2	0.97	0.01	134	0.94	1.00	345678
	1 mo	2	0.95	0.01	134	0.93	0.98	23456
	3 mo	2	0.94	0.01	134	0.92	0.97	23456
	6 mo	2	0.92	0.01	134	0.89	0.95	12
	12 mo	2	0.89	0.02	134	0.86	0.92	1
	baseline	3	1.01	0.01	134	0.98	1.04	4689
	1 mo	3	0.99	0.01	134	0.96	1.02	23456789
	3 mo	3	0.98	0.01	134	0.95	1.01	2345678
	6 mo	3	0.97	0.02	134	0.93	1.00	12357
	12 mo	3	0.97	0.02	134	0.93	1.00	12345678
	baseline	4	1.04	0.01	134	1.02	1.06	9
	1 mo	4	1.02	0.01	134	1.00	1.04	78
	3 mo	4	1.00	0.01	134	0.98	1.02	5678
	6 mo	4	0.98	0.01	134	0.96	1.00	23456
	12 mo	4	0.96	0.01	134	0.94	0.98	1234
Fat mass (DXA)	baseline	1	71484.00	6339.23	136	58947.79	84020.21	NA
	1 mo	1	64577.67	6339.23	136	52041.46	77113.88	NA
	3 mo	1	52697.46	6445.27	136	39951.54	65443.39	NA
	6 mo	1	41997.17	6339.23	136	29460.96	54533.38	NA
	12 mo	1	40037.27	6601.15	136	26983.10	53091.44	NA
	baseline	2	68278.04	2558.72	133	63216.99	73339.08	NA
	1 mo	2	60340.68	2552.77	133	55291.40	65389.95	NA
	3 mo	2	49832.87	2565.39	133	44758.63	54907.11	NA
	6 mo	2	41037.09	2614.07	133	35866.55	46207.62	NA
	12 mo	2	35889.03	2751.97	133	30445.74	41332.31	NA
	baseline	3	64970.71	2797.16	133	59438.04	70503.39	NA
	1 mo	3	57857.68	2788.89	133	52341.36	63373.99	NA
	3 mo	3	48762.01	2805.50	133	43212.85	54311.17	NA
	6 mo	3	41479.53	2859.86	133	35822.84	47136.22	NA
	12 mo	3	33804.26	3004.72	133	27861.05	39747.47	12
	baseline	4	64232.42	1962.35	133	60350.97	68113.86	NA
	1 mo	4	56814.92	1962.18	133	52933.80	60696.03	NA
	3 mo	4	46932.75	1964.69	133	43046.67	50818.83	NA
	6 mo	4	41327.89	1977.62	133	37416.23	45239.55	NA
	12 mo	4	36903.07	2050.27	133	32847.70	40958.43	135
Lean body mass (DXA)	baseline	1	76091.33	5020.04	136	66163.89	86018.78	NA
	1 mo	1	69184.50	5020.04	136	59257.06	79111.94	NA
	3 mo	1	68930.17	5020.04	136	59002.72	78857.61	1
	6 mo	1	67574.67	5020.04	136	57647.22	77502.11	1
	12 mo	1	67824.44	5077.98	136	57782.43	77866.46	1
	baseline	2	63309.78	2022.85	133	59308.67	67310.90	1
	1 mo	2	57526.70	2021.54	133	53528.17	61525.23	1234
	3 mo	2	57225.03	2025.89	133	53217.91	61232.16	1234
	6 mo	2	57082.43	2035.22	133	53056.85	61108.01	1234
	12 mo	2	57101.31	2066.61	133	53013.63	61188.98	1234
	baseline	3	66221.17	2210.35	133	61849.19	70593.15	23
	1 mo	3	59867.45	2208.53	133	55499.07	64235.83	125678
	3 mo	3	59416.80	2212.18	133	55041.19	63792.41	125678
	6 mo	3	58668.94	2224.28	133	54269.41	63068.48	125678

	12 mo	3	57440.70	2257.20	133	52976.04	61905.36	1256
	baseline	4	68246.66	1550.55	133	65179.73	71313.59	4
	1 mo	4	61629.61	1549.91	133	58563.94	64695.27	123
	3 mo	4	61120.43	1550.40	133	58053.80	64187.06	124
	6 mo	4	61066.98	1553.92	133	57993.39	64140.57	3
	12 mo	4	61005.23	1570.15	133	57899.54	64110.93	4
Physical activity	baseline	1	6468.25	3996.78	137	-1435.11	14371.61	123
	1 mo	1	2967.34	4296.96	137	-5529.61	11464.29	123
	3 mo	1	13212.83	3996.78	137	5309.48	21116.19	123
	6 mo	1	9390.40	4296.96	137	893.45	17887.35	123
	12 mo	1	12851.44	4712.62	137	3532.55	22170.33	123
	baseline	2	9713.04	1627.26	134	6494.61	12931.47	123
	1 mo	2	7852.02	1627.24	134	4633.62	11070.42	12
	3 mo	2	12073.64	1664.84	134	8780.88	15366.40	123
	6 mo	2	13114.97	1777.28	134	9599.82	16630.12	123
	12 mo	2	16769.66	2125.48	134	12565.83	20973.49	3
	baseline	3	8229.02	1758.35	134	4751.31	11706.72	123
	1 mo	3	7410.02	1758.35	134	3932.31	10887.72	123
	3 mo	3	10076.13	1806.43	134	6503.33	13648.93	123
	6 mo	3	10642.96	1987.61	134	6711.80	14574.11	123
	12 mo	3	15904.37	2317.03	134	11321.69	20487.05	23
	baseline	4	9308.51	1240.60	134	6854.82	11762.19	123
	1 mo	4	5905.48	1274.00	134	3385.73	8425.24	1
	3 mo	4	10977.83	1264.71	134	8476.45	13479.22	123
	6 mo	4	9821.70	1301.46	134	7247.64	12395.76	123
	12 mo	4	11520.95	1501.20	134	8551.84	14490.06	123
Mean insulin concentration during OGTT	baseline	1	111.50	20.17	137	71.61	151.39	1
	1 mo	1	22.21	20.17	137	-17.68	62.11	2
	3 mo	1	97.97	20.17	137	58.08	137.86	34
	6 mo	1	65.32	20.17	137	25.43	105.21	45
	12 mo	1	53.74	23.30	137	7.67	99.82	3
	baseline	2	95.62	8.12	134	79.55	111.68	70A
	1 mo	2	18.97	8.12	134	2.91	35.04	12
	3 mo	2	54.57	8.28	134	38.19	70.94	345689
	6 mo	2	47.79	8.86	134	30.26	65.31	12345689
	12 mo	2	37.41	10.40	134	16.84	57.99	123458
	baseline	3	130.69	8.87	134	113.14	148.24	A
	1 mo	3	20.60	8.97	134	2.85	38.35	13
	3 mo	3	84.29	9.19	134	66.10	102.47	567890
	6 mo	3	64.41	9.73	134	45.17	83.65	24567890
	12 mo	3	72.26	11.65	134	49.22	95.29	567890
	baseline	4	131.51	6.18	134	119.30	143.73	A
	1 mo	4	23.53	6.21	134	11.25	35.81	1234
	3 mo	4	85.07	6.24	134	72.73	97.42	6790
	6 mo	4	81.30	6.47	134	68.50	94.10	567890
	12 mo	4	64.00	7.32	134	49.53	78.48	567890
Mean glucose concentration during OGTT	baseline	1	114.04	10.02	137	94.23	133.85	123456
	1 mo	1	98.33	10.02	137	78.53	118.14	12345
	3 mo	1	122.13	10.02	137	102.32	141.93	123456
	6 mo	1	116.63	10.02	137	96.82	136.43	123456
	12 mo	1	111.13	11.63	137	88.14	134.13	123456

	baseline	2	136.18	4.03	134	128.20	144.16	56
	1 mo	2	101.35	4.03	134	93.37	109.33	1
	3 mo	2	114.71	4.11	134	106.57	122.85	1234
	6 mo	2	110.30	4.41	134	101.57	119.03	1234
	12 mo	2	108.97	5.21	134	98.67	119.27	1234
	baseline	3	163.71	4.41	134	154.99	172.43	7
	1 mo	3	108.52	4.46	134	99.71	117.34	13
	3 mo	3	126.67	4.51	134	117.74	135.60	2456
	6 mo	3	123.81	4.85	134	114.22	133.39	123456
	12 mo	3	119.40	5.68	134	108.17	130.63	123456
	baseline	4	199.03	3.07	134	192.96	205.09	8
	1 mo	4	114.64	3.08	134	108.54	120.74	12
	3 mo	4	139.51	3.10	134	133.37	145.64	6
	6 mo	4	136.22	3.20	134	129.89	142.54	56
	12 mo	4	128.81	3.62	134	121.65	135.97	3456
Matsuda index	baseline	1	1.62	1.43	137	-1.20	4.44	12345
	3 mo	1	3.14	1.43	137	0.32	5.96	12345678
	6 mo	1	5.90	1.43	137	3.08	8.72	12345678
	12 mo	1	8.51	1.67	137	5.21	11.81	678
	baseline	2	2.15	0.57	134	1.01	3.28	12
	3 mo	2	6.48	0.59	134	5.32	7.64	4578
	6 mo	2	6.82	0.63	134	5.57	8.07	58
	12 mo	2	7.20	0.75	134	5.71	8.68	58
	baseline	3	1.40	0.63	134	0.16	2.64	12
	3 mo	3	3.63	0.65	134	2.34	4.92	123467
	6 mo	3	5.83	0.69	134	4.46	7.20	345678
	12 mo	3	4.35	0.84	134	2.68	6.02	12345678
	baseline	4	1.13	0.44	134	0.26	1.99	1
	3 mo	4	3.22	0.44	134	2.34	4.09	236
	6 mo	4	3.58	0.46	134	2.67	4.49	236
	12 mo	4	5.10	0.53	134	4.06	6.14	345678
Glucose AUC	baseline	1	231.25	24.19	137	183.42	279.08	123
	3 mo	1	266.08	25.83	137	215.00	317.16	1234
	6 mo	1	280.86	30.89	137	219.77	341.95	1234
	12 mo	1	265.11	35.90	137	194.12	336.10	1234
	baseline	2	285.66	9.74	134	266.39	304.92	23
	3 mo	2	267.08	11.71	134	243.92	290.23	123
	6 mo	2	234.79	12.12	134	210.82	258.77	1
	12 mo	2	250.33	15.24	134	220.19	280.46	12
	baseline	3	353.06	10.64	134	332.01	374.10	4
	3 mo	3	273.83	11.45	134	251.17	296.48	123
	6 mo	3	279.21	12.58	134	254.32	304.10	123
	12 mo	3	259.84	14.01	134	232.13	287.56	123
	baseline	4	427.92	7.41	134	413.27	442.57	5
	3 mo	4	312.29	8.10	134	296.27	328.30	34
	6 mo	4	309.18	8.22	134	292.93	325.44	234
	12 mo	4	308.78	9.98	134	289.04	328.53	234
Insulin AUC	baseline	1	247.82	51.83	136	145.33	350.32	1234
	3 mo	1	278.52	55.10	136	169.56	387.48	1234
	6 mo	1	295.67	65.11	136	166.90	424.43	1234
	12 mo	1	223.20	75.06	136	74.76	371.64	1234

	baseline	2	227.33	21.16	133	185.48	269.18	1234
	3 mo	2	183.57	24.86	133	134.40	232.75	1
	6 mo	2	160.14	26.14	133	108.43	211.84	1
	12 mo	2	146.63	31.89	133	83.55	209.72	1
	baseline	3	309.44	22.80	133	264.34	354.54	34
	3 mo	3	216.19	24.41	133	167.90	264.47	12
	6 mo	3	183.46	27.10	133	129.86	237.07	1
	12 mo	3	181.85	29.47	133	123.56	240.15	12
	baseline	4	298.49	15.87	133	267.10	329.88	24
	3 mo	4	230.45	17.23	133	196.37	264.54	13
	6 mo	4	223.94	17.47	133	189.39	258.49	13
	12 mo	4	207.92	20.96	133	166.46	249.37	13

Table S6. Factor level tests on generalized linear mixed models comparing time points for OGTT-specific cross sections with predictors time + OGTT cluster + time × OGTT cluster, controlling for age, sex, and smoking status. Mean refers to model-adjusted mean; SE for standard error; df for degrees of freedom; LCL for the lower 95% confidence limit; UCL for the upper 95% confidence limit; CLD for compact letter display. Times-cluster combinations with different CLDs within a particular parameter indicates that they are different from each other. NAs represent failures in model convergence, and we do not attempt to speculate about post-hoc significances of the time-by-OGTT cluster factor level combinations for these levels.

	PC	cluster	LS mean	SE	df	LCL	UCL	CLD
Glucose homeostasis	PC1	4	-0.389	0.341	80	-1.068	0.290	1
		3	-0.007	0.533	80	-1.068	1.054	1
		2	0.475	0.519	80	-0.557	1.508	1
		1	2.735	1.306	80	0.136	5.333	1
	PC2	4	-0.579	0.216	80	-1.009	-0.149	1
		3	0.449	0.338	80	-0.223	1.121	12
		2	0.570	0.329	80	-0.084	1.224	2
		1	2.181	0.827	80	0.535	3.828	2
Anthropometric	PC1	1	-0.935	1.302	76	-3.527	1.657	1
		2	-0.666	0.631	76	-1.923	0.591	1
		4	0.062	0.407	76	-0.748	0.871	1
		3	0.696	0.614	76	-0.526	1.918	1
	PC2	3	-0.250	0.332	76	-0.911	0.412	1
		4	0.039	0.220	76	-0.399	0.478	1
		2	0.089	0.342	76	-0.591	0.770	1
		1	0.340	0.704	76	-1.063	1.743	1

Table S7. Post-hoc tests on generalized linear mixed models comparing principal component scores as "principal responses" to bariatric surgery among OGTT clusters controlling for age, sex, and smoking status. PC1 refers to principle component 1; PC2 refers to principle component 2. Cluster refers to OGTT cluster. LS mean refers to least square mean; SE for standard error; df for degrees of freedom; LCL for the lower 95% confidence limit; UCL for the upper 95% confidence limit; CLD for compact letter display. Clusters with different CLDs within a particular PC indicates that the response in those clusters are significantly different from each other.

model	FU	TPR	FPR	AUC
Model A: Hx	1 mo	0.433	0.163	0.699
	3 mo	0.539	0.099	0.720
	6 mo	0.234	0.073	0.740
	12 mo	0.488	0.301	0.609
Model B: Hx+OGTT	1 mo	0.500	0.059	0.897
	3 mo	0.571	0.071	0.908
	6 mo	0.333	0.077	0.885
	12 mo	0.800	0.222	0.844
Model C: Hx+OGTT+CRP+HbA1c	1 mo	1.000	0.000	1.000
	3 mo	0.667	0.143	0.917
	6 mo	0.600	0.154	0.892
	12 mo	1.000	0.000	1.000
Model D: Hx+OGTT+CRP+HbA1c+Rx	1 mo	1.000	0.000	1.000
	3 mo	1.000	0.000	1.000
	6 mo	1.000	0.000	1.000
	12 mo	1.000	0.000	1.000

Table S8. Nested model performance predicting diabetes improvement and remission from baseline parameters. The models contained the following baseline terms: Model A with patient history (Hx) variables (BMI, number of past years smoking, and years since diabetes diagnosis); Model B with patient history variables and OGTT cluster; Model C with patient history variables, OGTT cluster, C reactive protein concentration, and HbA1c; and Model D with patient history variables, OGTT cluster, C reactive protein concentration, HbA1c, and the number of medications prescribed to a patient one week prior to surgery considering only statins, metformin, insulin, gliptins, sulfonyl ureas, and glifozins. TPR is for true positive rate; FPR is for false positive rate, and AUC is for area under the curve.

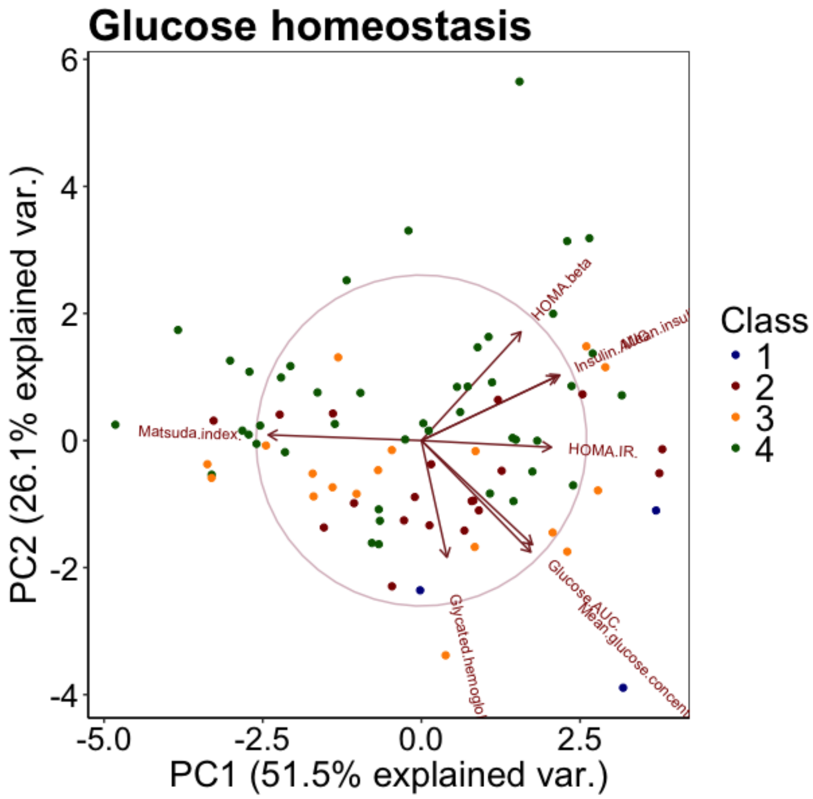

Figure S1. Biplot from principal components analysis of glucose homeostasis parameters (Table S1) with loadings plotted as arrows for each parameter and individual patients plotted as scores along PC1 and PC2. PC1 refers to the first principal component and PC2 refers to the second principal component; Cluster refers to OGTT cluster.

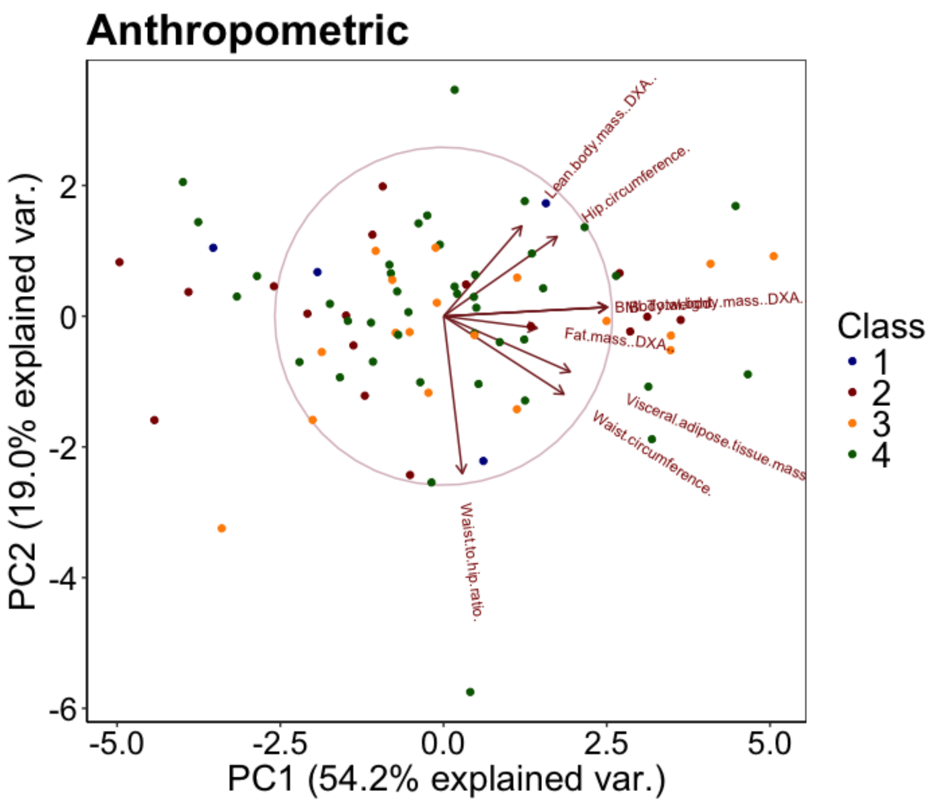

Figure S2. Biplot from principal components analysis of anthropometric parameters (Table S1) with loadings plotted as arrows for each parameter and individual patients plotted as scores along PC1 and PC2. PC1 refers to the first principal component and PC2 refers to the second principal component; Cluster refers to OGTT cluster.

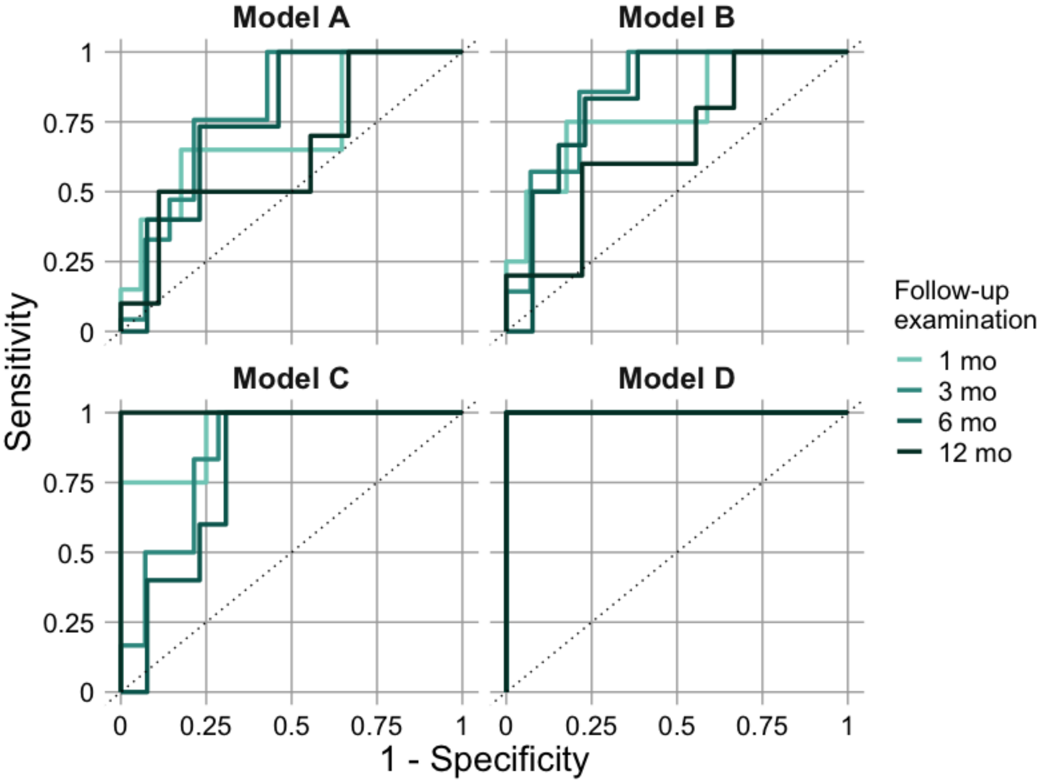

Figure S3. Receiver operating characteristic curves from multinomial regression predicting diabetes remission. These were fit sequentially to each follow-up exam visit when a patient was assessed for diabetes remission. The models contained the following baseline terms: Model A with patient history (Hx) variables (BMI, number of past years smoking, and years since diabetes diagnosis); Model B with patient history variables and OGTT cluster; Model C with patient history variables, OGTT cluster, C reactive protein concentration, and HbA1c; and Model D with patient history variables, OGTT cluster, C reactive protein concentration, HbA1c, and the number of medications prescribed to a patient one week prior to surgery considering only statins, metformin, insulin, gliptins, sulfonyl ureas, and glifozins.
